# Supplementary material for: Risk of placenta previa in assisted reproductive technology: A Nordic population study with sibling analyses
Source: PLoS Med. 2025 Feb 3;22(2):e1004536. doi: 10.1371/journal.pmed.1004536 (PMC11835333; doi:10.1371/journal.pmed.1004536)
Supplement: S1 Checklist — (DOCX) [file pmed.1004536.s001.docx]

The RECORD statement – checklist of items, extended from the STROBE statement, that should be reported in observational studies using routinely collected health data.

|  | **Item No.** | **STROBE items** | **Location in manuscript where items are reported** | **RECORD items** | **Location in manuscript where items are reported** |
| --- | --- | --- | --- | --- | --- |
| **Title and abstract** | | | | | |
|  | 1 | (a) Indicate the study’s design with a commonly used term in the title or the abstract (b) Provide in the abstract an informative and balanced summary of what was done and what was found | a) Title, abstract (methods and findings) and first section of methods.  b) Abstract (methods and findings). | RECORD 1.1: The type of data used should be specified in the title or abstract. When possible, the name of the databases used should be included.  RECORD 1.2: If applicable, the geographic region and timeframe within which the study took place should be reported in the title or abstract.  RECORD 1.3: If linkage between databases was conducted for the study, this should be clearly stated in the title or abstract. | 1.1-1.3) Abstract (methods and findings). |
| **Introduction** | | | | | |
| Background rationale | 2 | Explain the scientific background and rationale for the  investigation being reported | Introduction (first, second and third paragraph). |  |  |
| Objectives | 3 | State specific objectives, including any prespecified hypotheses | Introduction (fourth paragraph). |  |  |
| **Methods** | | | | | |
| Study Design | 4 | Present key elements of study design early in the paper | Method section (first, second, third and fourth paragraph). |  |  |
| Setting | 5 | Describe the setting, locations, and relevant dates, including  periods of recruitment, exposure, follow-up, and data collection | Method section (fifth and sixth paragraph). |  |  |

| Participants | 6 | 1. *Cohort study* - Give the eligibility criteria, and the sources and methods of selection of participants. Describe methods of follow-up   *Case-control study* - Give the eligibility criteria, and the sources and methods of case ascertainment and control selection. Give the rationale for the choice of cases and controls *Cross-sectional study* - Give the eligibility criteria, and the sources and methods of selection of participants   1. *Cohort study* - For matched studies, give matching criteria and number of exposed and unexposed   *Case-control study* - For matched studies, give matching criteria and the number of controls per case | a) Method section (fifth and sixth paragraph).  b) Not applicable. | RECORD 6.1: The methods of study population selection (such as codes or algorithms used to identify subjects) should be listed in detail. If this is not possible, an explanation should be provided.  RECORD 6.2: Any validation studies of the codes or algorithms used to select the population should be referenced. If validation was conducted for this study and not published elsewhere, detailed methods and results should be provided.  RECORD 6.3: If the study involved linkage of databases, consider use of a flow diagram or other graphical display to demonstrate the data linkage process, including the number of individuals with linked data at each stage. | 6.1) Method section (fifth and sixth paragraph).  6.2) Not applicable.  6.3) Fig 1. |
| --- | --- | --- | --- | --- | --- |
| Variables | 7 | Clearly define all outcomes, exposures, predictors, potential confounders, and effect modifiers. Give diagnostic criteria, if applicable. | Method section (second, third, fourth and seventh paragraph), S1-3 Tables. | RECORD 7.1: A complete list of codes and algorithms used to classify exposures, outcomes, confounders, and effect modifiers should be provided. If these cannot be reported, an explanation should be provided. | 7.1) Method section (second, third, fourth and seventh paragraph), S1-3 Tables. |
| Data sources/ measurement | 8 | For each variable of interest, give sources of data and details of methods of assessment (measurement).  Describe comparability of assessment methods if there is more than one group | Method section (second, third, fourth and seventh paragraph), S1-3 Tables. |  |  |

| Bias | 9 | Describe any efforts to address potential sources of bias | Method section (seventh, eight and ninth paragraph),  S1 Text. |  |  |
| --- | --- | --- | --- | --- | --- |
| Study size | 10 | Explain how the study size was arrived at | Method section (fifth and sixth paragraph), Fig 1. |  |  |
| Quantitative variables | 11 | Explain how quantitative variables were handled in the analyses. If applicable, describe  which groupings were chosen, and why | Method section (seventh paragraph), Table legends (Tables 2 and 3, S4-6 Tables),  Figure legends (Figs 2 and 3) |  |  |
| Statistical methods | 12 | 1. Describe all statistical methods, including those used to control for confounding 2. Describe any methods used to examine subgroups and interactions 3. Explain how missing data were addressed 4. *Cohort study* - If applicable, explain how loss to follow-up was addressed   *Case-control study* - If applicable, explain how matching of cases and controls was addressed  *Cross-sectional study* - If applicable, describe analytical methods taking account of sampling strategy   1. Describe any sensitivity analyses | a) Method section (seventh, eight and ninth paragraph),  S1 Text,  Table legends (Table 2 and 3, S4-6 Tables),  Figure legends (Figs 2-3).  b) Method section (seventh, eight and ninth paragraph), Figure legends (Figs 2 and 3), Table legends (S4-6 Tables),  S1 Text.  c) Method section (fifth and sixth paragraph), Fig 1.  d) Not applicable.  e) Method section (seventh, eight and ninth paragraph), Figure legends (Figs 2 and 3), Table legends (S4-6 Tables),  S1 Text. |  |  |
| Data access and cleaning methods |  | .. |  | RECORD 12.1: Authors should describe the extent to which the investigators had access to the database population used to create the study population. | 12.1) Data availability statement. |

|  |  |  |  | RECORD 12.2: Authors should provide information on the data cleaning methods used in the study. | 12.2) Method section (second, third and fourth paragraph). |
| --- | --- | --- | --- | --- | --- |
| Linkage |  | .. |  | RECORD 12.3: State whether the study included person-level, institutional-level, or other data linkage across two or more databases. The methods of linkage and methods of  linkage quality evaluation should be provided. | 12.3) Method section (first paragraph). |
| **Results** | | | | | |
| Participants | 13 | 1. Report the numbers of individuals at each stage of the study (*e.g.*, numbers potentially eligible, examined for eligibility, confirmed eligible, included in the study, completing follow-up, and analysed) 2. Give reasons for non- participation at each stage. 3. Consider use of a flow diagram | a-c) Fig 1. | RECORD 13.1: Describe in detail the selection of the persons included in the study (*i.e.,* study population selection) including filtering based on data quality, data availability and linkage. The selection of included persons can be described in the text and/or by means of the study flow diagram. | 13.1) Method section (fifth and sixth paragraph), Fig 1. |
| Descriptive data | 14 | 1. Give characteristics of study participants (*e.g.*, demographic, clinical, social) and information on exposures and potential confounders 2. Indicate the number of participants with missing data for each variable of interest 3. *Cohort study* - summarise follow-up time (*e.g.*, average and total amount) | a) Result section (first paragraph).  b) Table Legend (S6 Table).  c) Not applicable. |  |  |
| Outcome data | 15 | *Cohort study* - Report numbers of outcome events or summary measures over time  *Case-control study* - Report numbers in each exposure | Tables (Tables 2 and 3, S4-6 Tables). |  |  |

|  |  | category, or summary measures of exposure  *Cross-sectional study* - Report numbers of outcome events or summary measures |  |  |  |
| --- | --- | --- | --- | --- | --- |
| Main results | 16 | 1. Give unadjusted estimates and, if applicable, confounder- adjusted estimates and their precision (e.g., 95% confidence interval). Make clear which confounders were adjusted for and why they were included 2. Report category boundaries when continuous variables were categorized 3. If relevant, consider translating estimates of relative risk into absolute risk for a meaningful time period | a) Result section (second, third, fourth, fifth and sixth paragraph),  Tables (Tables 2 and 3, S4-6 Tables),  Figs 2 and 3.  b) Method section (seventh paragraph), Table legends (Tables 2 and 3, S4-6 Tables,  Figure legends (Figs 2 and 3).  c) Fig 2. |  |  |
| Other analyses | 17 | Report other analyses done— e.g., analyses of subgroups and interactions, and sensitivity analyses | Figs 2-3),  Tables (S4 and S5 Tables). |  |  |
| **Discussion** | | | | | |
| Key results | 18 | Summarise key results with  reference to study objectives | Discussion section (first paragraph). |  |  |
| Limitations | 19 | Discuss limitations of the study, taking into account sources of potential bias or imprecision.  Discuss both direction and magnitude of any potential bias | Discussion section (ninth, tenth, eleventh and twelfth paragraph). | RECORD 19.1: Discuss the implications of using data that were not created or collected to answer the specific research question(s). Include discussion of misclassification bias, unmeasured confounding, missing data, and changing eligibility over time, as they pertain to the study being  reported. | Discussion section (ninth, tenth and eleventh paragraph). |
| Interpretation | 20 | Give a cautious overall interpretation of results considering objectives, |  |  | Discussion section (thirteenth paragraph). |

|  |  | limitations, multiplicity of analyses, results from similar studies, and other relevant evidence |  |  |  |
| --- | --- | --- | --- | --- | --- |
| Generalisability | 21 | Discuss the generalisability (external validity) of the study results |  |  | Discussion section (twelfth paragraph). |
| **Other Information** | | | | | |
| Funding | 22 | Give the source of funding and the role of the funders for the present study and, if applicable, for the original study on which the present article is based | Funding. |  |  |
| Accessibility of protocol, raw data, and programming  code |  | .. |  | RECORD 22.1: Authors should provide information on how to access any supplemental information such as the study protocol, raw data, or  programming code. | Data availability statement. |

*Reference: Benchimol EI, Smeeth L, Guttmann A, Harron K, Moher D, Petersen I, Sørensen HT, von Elm E, Langan SM, the RECORD Working Committee. The REporting of studies Conducted using Observational Routinely-collected health Data (RECORD) Statement. *PLoS Medicine* 2015; in press.

*Checklist is protected under Creative Commons Attribution ([CC BY](http://creativecommons.org/licenses/by/4.0/)) license.
