# Supplementary material for: Risk of placenta previa in assisted reproductive technology: A Nordic population study with sibling analyses
Source: PLoS Med. 2025 Feb 3;22(2):e1004536. doi: 10.1371/journal.pmed.1004536 (PMC11835333; doi:10.1371/journal.pmed.1004536)
Supplement: S1 Table — (DOCX) [file pmed.1004536.s002.docx]

| **S1 Table.** Data sources and registration practice for placenta previa in the Nordic countries during the study period. | | | | |
| --- | --- | --- | --- | --- |
|  | Country | | | |
|  | Denmark | Finland | Norway | Sweden |
| Medical Birth Registry | - | 2004-2014 | 1988-2015 | 1988-2015 |
| Registration practice | - | ICD codes | Text field^a^ <1999 | ICD codes |
|  |  |  | Tick box ≥1999 |  |
| National Patient Registry | 1994-2014 | 1990-2014 | - | - |
| Registration practice | ICD codes | ICD codes | - | - |
| Classification system |  |  |  |  |
| ICD-8: 632.0 | - | - | 1988–1998 | - |
| ICD-9: 641.0/1/A/B | - | 1990-1995 | - | 1988-1996 |
| ICD-10: O44.0/1 | 1994-2014 | 1996-2014 | 1999-2015 | 1997-2015 |
| ^a^Open text field where the reporter was free to put ICD-codes or plain text. In the case of plain text, data were recoded as ICD-codes by the registry holder. | | | | |
