# Supplementary material for: Risk of placenta previa in assisted reproductive technology: A Nordic population study with sibling analyses
Source: PLoS Med. 2025 Feb 3;22(2):e1004536. doi: 10.1371/journal.pmed.1004536 (PMC11835333; doi:10.1371/journal.pmed.1004536)
Supplement: S2 Table — (DOCX) [file pmed.1004536.s003.docx]

| **S2 Table.** Data sources and registration practice for infertility factors in the Nordic countries during the study period. | | | | |
| --- | --- | --- | --- | --- |
|  | Country | | | |
|  | Denmark | Finland | Norway | Sweden |
| Medical Birth Registry | - | - | 1988-2015 | - |
| Registration practice | - | - | Tick box | - |
|  |  |  |  |  |
| National Patient Registry | 1994-2014 | 1987-2014 | 2008-2015 | 1987-2015 |
| Registration practice | ICD codes | ICD codes | ICD codes^a^ | ICD codes |
| National IVF registry | 1994-2014 | - | - | - |
| Registration practice | Tick box <2006 | - | - | - |
|  | ICD codes ≥2006 |  |  |  |
| Classification system |  |  |  |  |
| ICD-9 | - | 1987-1995 | - | 1987-1996 |
| ICD-10 | 1994-2014 | 1996-2014 | 2008-2015 | 1997-2015 |
| ^a^In Norway, the National Patient Registry only provided diagnoses of polycystic ovary syndrome. | | | | |
