# Supplementary material for: Risk of placenta previa in assisted reproductive technology: A Nordic population study with sibling analyses
Source: PLoS Med. 2025 Feb 3;22(2):e1004536. doi: 10.1371/journal.pmed.1004536 (PMC11835333; doi:10.1371/journal.pmed.1004536)
Supplement: S3 Table — (DOCX) [file pmed.1004536.s004.docx]

| **S3 Table.** ICD codes used for identification of infertility factors in the Nordic countries during the study period. | | |  |
| --- | --- | --- | --- |
|  | ICD-9 | ICD-10 | |
| Uterine factors | 628.3, 628D | N97.2 | |
| Endometriosis | 617 | N80 | |
| Polycystic ovary syndrome | 256.4, 256E | E28.2 | |
| Male factors | 606 | N97.4, N46 | |
| Other infertility factors | 628.0, 628.1, 628.2, 628.4, 628.8, 628.9, 628A, 628B, 628C, 628E, 628X, 628W | N97.0, N97.1, N97.3, N97.8, N97.9 | |
