# Supplementary material for: Risk of placenta previa in assisted reproductive technology: A Nordic population study with sibling analyses
Source: PLoS Med. 2025 Feb 3;22(2):e1004536. doi: 10.1371/journal.pmed.1004536 (PMC11835333; doi:10.1371/journal.pmed.1004536)
Supplement: S4 Table — (DOCX) [file pmed.1004536.s005.docx]

| **S4 Table.** Observed risk and adjusted odds ratio of placenta previa in second pregnancy according to conception method and caesarean section in first pregnancy in sample 1. | | | | | |
| --- | --- | --- | --- | --- | --- |
| Conception and delivery method in first pregnancy | Conception method in second pregnancy | | | Risk of placenta previa in second birth, ART vs NC^a^ | |
|  |  | 5-year follow-up^b^, n (%) | Full study period, n (%) | Observed risk | Adjusted odds ratio^c^ (95% CI) |
| NC conception -no caesarean section | NC pregnancy | 1,508,347 (79.3) | 1,655,388 (70.2) | 3.2/1000 | 1 (ref) |
|  | ART pregnancy | 10,796 (0.6) | 11,408 (0.5) | 21.9/1000 | 4.73 (4.15 to 5.40) |
|  | No continuation | 383,590 (20.2) | 690,906 (29.3) |  |  |
| NC conception -caesarean section | NC pregnancy | 266,066 (67.6) | 295,022 (58.4) | 5.8/1000 | 1 (ref) |
|  | ART pregnancy | 2933 (0.8) | 3141 (0.6) | 29.0/1000 | 3.62 (2.91 to 4.50) |
|  | No continuation | 124,431 (31.6) | 206,644 (40.9) |  |  |
| ART conception -no caesarean section | NC pregnancy | 13,693 (29.4) | 16,611 (24.4) | 7.9/1000 | 1 (ref) |
|  | ART pregnancy | 12,059 (25.9) | 14,658 (21.5) | 16.6/1000 | 2.06 (1.66 to 2.56) |
|  | No continuation | 20,775 (44.7) | 36,856 (54.1) |  |  |
| ART conception -caesarean section | NC pregnancy | 4767 (20.6) | 5731 (17.5) | 12.0/1000 | 1 (ref) |
|  | ART pregnancy | 3598 (15.5) | 4310 (13.2) | 30.2/1000 | 2.59 (1.92 to 3.49) |
|  | No continuation | 14,812 (63.9) | 22,691 (69.3) |  |  |
| Abbreviations: ART, assisted reproductive technology; NC, natural conception; CI, confidence interval. | | | | | |
| ^a^Results are presented for the full study period. | | | | | |
| ^b^Observed proportions are presented for mothers with at least five years of follow-up after first delivery. | | | | | |
| ^c^Adjusted for year of delivery (categorical: 1988–1996, 1997–2001, 2002–2006, 2007–2011, 2012–2015), maternal age (categorical: 20–24, 25–29, 30–34, 35–39, 40–45), parity (categorical: 0, 1, 2, 3) and country. | | | | | |
