# Supplementary material for: Risk of placenta previa in assisted reproductive technology: A Nordic population study with sibling analyses
Source: PLoS Med. 2025 Feb 3;22(2):e1004536. doi: 10.1371/journal.pmed.1004536 (PMC11835333; doi:10.1371/journal.pmed.1004536)
Supplement: S5 Table — (DOCX) [file pmed.1004536.s006.docx]

| **S5 Table.** Observed risk and adjusted odds ratio of placenta previa in second pregnancy according to conception method and placenta previa in first pregnancy in sample 1. | | | | | |
| --- | --- | --- | --- | --- | --- |
| Conception and delivery method in first pregnancy | Conception method in second pregnancy | | | Risk of placenta previa in second birth, ART vs NC^a^ | |
|  |  | 5-year follow-up^b^, n (%) | Full study period, n (%) | Observed risk | Adjusted odds ratio^c^ (95% CI) |
| NC conception -no placenta previa | NC pregnancy | 1,770,912 (77.3) | 1,946,430 (68.2) | 3.5/1000 | 1 (ref) |
|  | ART pregnancy | 13,660 (0.6) | 14,471 (0.5) | 22.9/1000 | 4.41 (3.93 to 4.94) |
|  | No continuation | 506,206 (22.1) | 894,382 (31.3) |  |  |
| NC conception -placenta previa | NC pregnancy | 3501 (65.0) | 3980 (55.1) | 34.4/1000 | 1 (ref) |
|  | ART pregnancy | 69 (1.3) | 78 (1.1) | 128.2/1000 | 3.34 (1.66 to 6.72) |
|  | No continuation | 1815 (33.7) | 3168 (43.8) |  |  |
| ART conception -no placenta previa | NC pregnancy | 18,207 (26.5) | 22,012 (22.2) | 8.4/1000 | 1 (ref) |
|  | ART pregnancy | 15,459 (22.5) | 18,724 (18.9) | 18.9/1000 | 2.25 (1.88 to 2.69) |
|  | No continuation | 35,058 (51.0) | 58,529 (59.0) |  |  |
| ART conception -placenta previa | NC pregnancy | 253 (25.8) | 330 (20.7) | 45.5/1000 | 1 (ref) |
|  | ART pregnancy | 198 (20.2) | 244 (15.3) | 77.9/1000 | 1.74 (0.85 to 3.55) |
|  | No continuation | 529 (54.0) | 1018 (63.9) |  |  |
| Abbreviations: ART, assisted reproductive technology; NC, natural conception; CI, confidence interval. | | | | | |
| ^a^Results are presented for the full study period. | | | | | |
| ^b^Observed proportions are presented for mothers with at least five years of follow-up after first delivery. | | | | | |
| ^c^Adjusted for year of delivery (categorical: 1988–1996, 1997–2001, 2002–2006, 2007–2011, 2012–2015), maternal age (categorical: 20–24, 25–29, 30–34, 35–39, 40–45), parity (categorical: 0, 1, 2, 3) and country. | | | | | |
