# Supplementary material for: Risk of placenta previa in assisted reproductive technology: A Nordic population study with sibling analyses
Source: PLoS Med. 2025 Feb 3;22(2):e1004536. doi: 10.1371/journal.pmed.1004536 (PMC11835333; doi:10.1371/journal.pmed.1004536)
Supplement: S6 Table — (DOCX) [file pmed.1004536.s007.docx]

| **S6 Table.** Description of pregnancies with and without placenta previa according to conception method and plurality in sample 1. | | | | |
| --- | --- | --- | --- | --- |
|  | ART | | NC | |
|  | Previa | No previa | Previa | No previa |
| Singleton pregnancies |  |  |  |  |
| Total number of pregnancies, n | 2144 | 117,447 | 17,339 | 5,523,491 |
| Mean gestational age, days (SD) | 259.0 (18.8) | 276.9 (15.6) | 260.8 (21.5) | 278.9 (13.0) |
| Preterm birth^a^, % | 36.3 | 7.5 | 35.6 | 4.8 |
| Very preterm birth^b^, % | 5.5 | 1.5 | 6.0 | 0.7 |
| Mean birthweight^c^, g (SD) | 2986 (669) | 3447 (623) | 3045 (732) | 3537 (564) |
| Mean birthweight z-score^d^ (SD) | 0.0 (1.2) | -0.1 (1.2) | 0.0 (1.3) | 0.0 (1.2) |
| SGA^e^, % | 4.2 | 4.8 | 4.6 | 3.7 |
| LGA^f^, % | 4.5 | 4.2 | 4.7 | 4.5 |
| Caesarean section, % | 90.4 | 25.1 | 77.6 | 15.2 |
| Twin pregnancies |  |  |  |  |
| Total number of pregnancies, n | 253 | 19,260 | 267 | 72,237 |
| Mean gestational age, days (SD) | 244.1 (20.4) | 253.6 (21.5) | 241.1 (25.1) | 254.6 (21.2) |
| Preterm birth^a^, % | 72.3 | 45.8 | 70.4 | 44.2 |
| Very preterm birth^b^, % | 17.0 | 8.8 | 20.2 | 8.3 |
| Mean birthweight^c^, g (SD) | 2295 (610) | 2501 (638) | 2204 (727) | 2519 (634) |
| Mean birthweight z-score^d^ (SD) | -0.9 (1.5) | -1.1 (1.5) | -1.1 (1.3) | -1.1 (1.2) |
| SGA^e^, % | 18.3 | 19.9 | 20.5 | 20.1 |
| LGA^f^, % | 1.4 | 0.6 | 0.8 | 0.5 |
| Caesarean section, % | 92.5 | 58.0 | 86.5 | 49.5 |
| Abbreviations: ART, assisted reproductive technology; NC, natural conception; Previa, Placenta previa; SD, standard deviation; SGA, small for gestational age; LGA, large for gestational age. | | | | |
| ^a^Gestational age <37 weeks. | | | | |
| ^b^Gestational age <32 weeks. | | | | |
| ^c^Data on birthweight were available for >99.2% in each category. | | | | |
| ^d^Birthweight z-score is defined as number of standard deviations from expected birthweight according to gestational age in days and sex. It was calculated using Marsal’s formulas, with one standard deviation equal to 11% [1]. | | | | |
| ^e^Birthweight z-score <-2. | | | | |
| ^f^Birthweight z-score >2. | | | | |

## **References**

1. Marsál K, Persson PH, Larsen T, Lilja H, Selbing A, Sultan B. Intrauterine growth curves based on ultrasonically estimated foetal weights. Acta Paediatr. 1996;85: 843–848. doi:10.1111/j.1651-2227.1996.tb14164.x
