# Supplementary material for: Risk of placenta previa in assisted reproductive technology: A Nordic population study with sibling analyses
Source: PLoS Med. 2025 Feb 3;22(2):e1004536. doi: 10.1371/journal.pmed.1004536 (PMC11835333; doi:10.1371/journal.pmed.1004536)
Supplement: S1 Text — (DOCX) [file pmed.1004536.s008.docx]

**S1 Text**

**More detailed description of sensitivity analyses**

We conducted sensitivity analyses in various subpopulations and models. In sample 1, analyses of placenta previa in ART vs NC was restricted to each woman’s second pregnancy and stratified according to conception method and outcome (caesarean section or placenta previa) in the first pregnancy. We further evaluated whether caesarean section or placenta previa in the first pregnancy influenced the need of ART in subsequent pregnancy (carryover effect) by describing the probability of ART in the second pregnancy for each combination of conception method and outcome (caesarean section or placenta previa) in the first pregnancy [1].

We did a series of sensitivity analyses in sample 3, where we had information on both embryo cryopreservation and culture duration. First, we restricted the diagnosis of placenta previa to pregnancies delivered by caesarean section to better capture cases with complete obstruction of the birth canal at delivery. Second, we repeated analyses in six subpopulations: only pregnancies conceived by the same couple (i.e., full siblings), each mother’s first two consecutive deliveries, only consecutive deliveries less than three years apart, only ART-conceived pregnancies after single embryo transfers (using all deliveries after NC as reference), only ART-conceived pregnancies after fertilization by IVF (using all deliveries after NC as reference), and only singleton pregnancies. Third, we repeated analyses in a subpopulation with available information on smoking status and added smoking status as a categorical covariate (yes, no) in the multilevel logistic regression model.

## **References**

1. Sjölander A, Frisell T, Kuja-Halkola R, Öberg S, Zetterqvist J. Carryover Effects in Sibling Comparison Designs. Epidemiology. 2016;27: 852–858. doi:10.1097/EDE.0000000000000541
