# Supplementary material for: Risk of placenta previa in assisted reproductive technology: A Nordic population study with sibling analyses
Source: PLoS Med. 2025 Feb 3;22(2):e1004536. doi: 10.1371/journal.pmed.1004536 (PMC11835333; doi:10.1371/journal.pmed.1004536)
Supplement: S1 Study Protocol — (PDF) [file pmed.1004536.s009.pdf]

Risk of Placenta Previa (and placenta accreta) in Assisted Reproduction: A Nordic Registry-based Population Study with Within-sibship Analyses (CoNARTaS)

E. Landsverk[1], K. Westvik-Johari[1,2] and S. Opdahl[1] with the Committee of Nordic Assisted Reproductive Technology and Safety (CoNARTaS).

Author affiliations

[1]*Department of Public Health and Nursing, Faculty of Medicine and Health Science, Norwegian University of Science and Technology, Trondheim, Norway*

[2]*Department of Fertility, Division of Obstetrics and Gynecology, St. Olavs Hospital, Trondheim University Hospital, Trondheim, Norway*

1 Background and aims

The use of assisted reproduction (ART) is increasing worldwide[1]. In the Nordic countries alone, more than 13 000 children (5%) are now born annually after ART[2–5]. A meta-analysis from 2018 indicate increased risk of placenta previa after ART[6], which is of concern as maternal complications secondary to placenta previa include caesarean section, antepartum and postpartum bleeding[7–11], in addition to increased risk of neonatal complications such as preterm birth and neonatal death[12,13]. There has been an increasing risk of placenta previa over time in Nordic ART-pregnancies[14] . To what extent this trend is driven by changes in how ART is performed is unclear and twin pregnancies do not seem to be a clear risk factor (Sindre, Oberg).

Separating the contributions of patient characteristics from treatment per se is possible with a sibling design, where pregnancies conceived after ART are compared with their naturally conceived pregnancies (NC). An early study from Norway found an increased risk of placenta previa in ART pregnancies compared to their naturally conceived pregnancies,[15] but did not differentiate between different ART-methods. More recent studies have linked fresh embryo transfer (fresh-ET) and blastocyst transfer to increased risk of placenta previa[16–20], with a particular high risk after fresh blastocyst transfer, indicating an interaction between fresh-ET and blastocyst transfer[16]. Using a large study population, we aim to investigate the risk of placenta previa after different treatment methods in a sibling study design.

2 Outline of methods

In this study we will use the CoNARTaS cohort, which is a population-based cohort of data from national health registries in Denmark (1994-2014), Finland (1990-2014), Norway (1984-2015) and Sweden (1985-2015). Mothers' unique national identity is used for linkage across registries[21].

2.1 Data sources and variables

ART pregnancies are identified through a cycle-based ART-registry in Denmark, a national registry of deliveries after ART (before 2007) and a cycle-based ART registry (from 2007) in Sweden, and Medical Birth Registries in Norway and Finland. Exposure will be conception by ART, defined as any pregnancy conceived outside the female body, i.e. fertilization with IVF or ICSI. Information on ART conception is available from all four countries. Pregnancies not registered as ART conception, will be considered as naturally conceived and will be used as the reference. ART will be further categorized according to cryopreservation status (fresh embryo transfer or frozen embryo transfer) and culture duration (cleavage stage or blastocyst) in Denmark, Norway and Sweden, where such information is available.

The outcome, placenta previa, will be identified as all cases registered in the Medical Birth Registry of Finland, Norway and Sweden, and diagnoses during the third trimester or within one month before delivery in the National Patient Registries of Denmark and Finland, as described in Table 1.

Table 1. Data sources and registration practice for placenta previa in the Nordic countries during the study period

	Country			
	Denmark	Finland	Norway	Sweden
Medical Birth Registry	-	2004-2014	1988-2015	1988-2015
Registration practice	-	ICD codes	Text field <1999<br>Tick box 1999->	ICD codes
National Patient Registry	1994-2014	1990-2014	-	-
Registration practice	ICD codes	ICD codes	-	-
Classification system				
ICD-8: 632.0	-	-	1988–1998	-
ICD-9: 641.0/1	-	1989-1995	-	1988-1996
ICD-10: O44	1994-2014	1996-2014	1999-2015	1997-2015

2.3 Study population:

The study population will be deliveries during 1994-2014 in Denmark, 1990-2014 in Finland, and 1988-2015 in Norway and Sweden. Exclusion of the first years in Norway and Sweden is due to very few deliveries after ART overall, and none after frozen-ET before 1988. We will define several analysis samples according to availability of exposure details.

Sample 1 will include all countries, with any ART conception as the exposure. All pregnancies, including multiple gestations, by mothers with their first delivery within study period and at age ≥ 20 will be eligible. This ensures a comparable range of year of delivery and maternal age between ART- and NC-pregnancies. Pregnancies with missing maternal age, birth order (parity) and gestational age are excluded. Maternal age > 45 years, and birth order ≥ 5, are excluded as there are extremely few ART-mothers with these characteristics. In addition, extreme values of gestational age are excluded (<22 weeks or > 44 weeks).

Sample 2 and 3 will consist of data from Denmark, Norway and Sweden, where ART-pregnancies with missing cryopreservation status are excluded from sample 1 to generate sample 2, and ART-pregnancies with missing culture duration are excluded from sample 2 to generate sample 3.

2.4 Statistical analyses:

We plan to estimate crude and adjusted odds ratios (ORs) for placenta previa at the population level and within sibships using multilevel logistic regression analyses where pregnancies are nested within mothers. For estimates at the population level, we will use random intercept models where each mother contributes 1-4 pregnancies. Within sibship estimates are obtained using a fixed intercept model (also referred to as a conditional model)[22] where each mother contributes with 2-4 pregnancies. Only pregnancies with both discordant exposure status and discordant outcome ("double discordant") contribute to the final exposure-outcome estimate in the fixed intercept model. Available data on factors that affect the need for ART and the risk of placenta previa (i.e., confounders) are included as covariates in the regression model; maternal age, birth order, birth year and country. To facilitate interpretation of the results we will use postestimation commands to calculate the absolute risk differences.

To test the robustness of the results, we will repeat the analyses in various subpopulations and models. First, we will include only singleton pregnancies. Second, we will include only pregnancies with information on smoking and repeat analyses with smoking added (as a categorical covariate; yes, no) to the logistic regression model. Third, we will include only consecutive pregnancies less than three years apart, as pregnancies within each mother have more in common when the interval is shorter between each pregnancy. Fourth, we will include only pregnancies conceived by the same couple (i.e. same father, full siblings) as these pregnancies might be more comparable. Fifth, we will restrict ART conceived pregnancies to those conceived after intracytoplasmic sperm injection. Sixth, we will restrict ART conceived pregnancies to those conceived after conventional in vitro fertilization. Seventh, we will restrict ART conceived pregnancies to those conceived after single embryo transfer. Eighth, we will redefine the outcome to placenta previa with caesarean section to better capture the cases with complete obstruction of the birth channel at delivery. Ninth, we will restrict sample to mothers' first two consecutive pregnancies to avoid overrepresentation of pregnancies from women that are more likely to proceed with a third and fourth pregnancy such as those with less severe complications in previous pregnancies.

To evaluate whether placenta previa in the first pregnancy influences the need for ART in subsequent pregnancy (carryover effect), we will describe the probability of ART in the second pregnancy for each combination of conception method (ART or natural conception) and outcome in the first pregnancy (i.e., ART – placenta previa, ART – no placenta previa, NC – placenta previa, NC – no placenta previa). Further, we will examine the risk of placenta previa in the second pregnancy with fixed exposure and outcome in the first pregnancy. In the same way, we will investigate carryover effect of caesarean section, and estimate risk of placenta previa in the second pregnancy between women with the same history of conception method and caesarean section.

To check whether the risk of placenta previa within sibships are driven by specific combinations of birth order and exposure, we will repeat logistic regression analyses with an additional interaction term between birth order and the nine combination of exposure status and birth order (i.e. NC – NC, NC – fresh-ET, NC – frozen-ET, fresh-ET – NC, fresh-ET – fresh-ET, fresh-ET – frozen-ET, frozen-ET – NC, frozen-ET – fresh-ET, frozen-ET – frozen-ET) for the

mothers' first and second delivery. Depending on the number of observations in each group, we will consider presenting such bidirectional analyses also according to culture duration.

We will examine the association between underlaying cause of infertility, ART and the risk of placenta previa in a subpopulation of pregnancies with registered cause of infertility. In addition, we will examine the association between twin pregnancies, ART and placenta previa (interaction between conception method and plurality presented as predicted risk in 2x3 table, where we divide twin pregnancies into same-sex and opposite sex).

Statistical power will be most limited for within sibship analyses in main sample 2 and 3 which are displayed in table 2.

Table 2. Expected number of discordant sibling groups in the study population, with corresponding odds ratios that may be detected with 80% power and 5% alpha level according to conception method and baseline risk of placenta previa of 0.5% after natural conception.

	**Main sample 2**		**Main sample 3**			
			**Cleavage**		**Blastocyst**	
ART type	**Fresh**	**Frozen**	**Fresh**	**Frozen**	**Fresh**	**Frozen**
Discordant sibships (n)	35 000	10 000	27 000	7000	2300	1700
Detectable odds ratio	1.3	1.6	1.4	1.8	2.5	2.8

3 References

1. Lancaster, P. & de Mouzon, J. Global Committee reproductive art surveillance: monitoring technologies assisted the (ICMART) International. *Assisted Reproductive Technology Surveillance* **101**, (2019).
2. Sundhedsdatastyrelsen. Assisteret reproduktion - Sundhedsdatastyrelsen. https://sundhedsdatastyrelsen.dk/da/find-tal-og-analyser/tal-og-analyser/sygdomme-og-behandlinger/assisteret-reproduktion.
3. Norwegian Institute of Public Health. Medisinsk fødselsregister - statistikkbank. https://statistikkbank.fhi.no/mfr/.
4. Nationellt kvalitetsregister för assisterad befruktning. Q-IVF — Årsrapporter. https://www.medscinet.com/qivf/arsrapporter.aspx.

5. Finnish institute for health and welfare. Assested fertility treatments - THL User Interface for Database Cubes and Reports. https://sampo.thl.fi/pivot/prod/en/ivf/ivfpublic/fact_ivfpublic.
6. Karami, M., Jenabi, E. & Fereidooni, B. The association of placenta previa and assisted reproductive techniques: a meta-analysis. *The Journal of Maternal-Fetal & Neonatal Medicine* **31**, 1940–1947 (2018).
7. Crane, J. M., Van den Hof, M. C., Dodds, L., Armson, B. A. & Liston, R. Maternal complications with placenta previa. *Am J Perinatol* **17**, 101–105 (2000).
8. Onwere, C. *et al.* Maternal morbidity associated with placenta praevia among women who had elective caesarean section. *European Journal of Obstetrics & Gynecology and Reproductive Biology* **159**, 62–66 (2011).
9. Lal, A. K. & Hibbard, J. U. Placenta previa: an outcome-based cohort study in a contemporary obstetric population. *Arch Gynecol Obstet* **292**, 299–305 (2015).
10. Fan, D. *et al.* The Incidence of Postpartum Hemorrhage in Pregnant Women with Placenta Previa: A Systematic Review and Meta-Analysis. *PLoS One* **12**, e0170194 (2017).
11. GIBBINS, K. J., EINERSON, B. D., VARNER, M. W. & SILVER, R. M. Placenta Previa and Maternal Hemorrhagic Morbidity. *J Matern Fetal Neonatal Med* **31**, 494–499 (2018).
12. Salihu, H. M., Li, Q., Rouse, D. J. & Alexander, G. R. Placenta previa: neonatal death after live births in the United States. *Am J Obstet Gynecol* **188**, 1305–1309 (2003).
13. Vahanian, S. A., Lavery, J. A., Ananth, C. V. & Vintzileos, A. Placental implantation abnormalities and risk of preterm delivery: a systematic review and metaanalysis. *Am J Obstet Gynecol* **213**, S78-90 (2015).
14. Petersen, S. H. *et al.* Time trends in placenta-mediated pregnancy complications after assisted reproductive technology in the Nordic countries. *Am J Obstet Gynecol* **223**, 226.e1-226.e19 (2020).

15. Romundstad, L. B. *et al.* Increased risk of placenta previa in pregnancies following IVF/ICSI; a comparison of ART and non-ART pregnancies in the same mother. *Hum Reprod* **21**, 2353–2358 (2006).
16. Spangmose, A. L. *et al.* Obstetric and perinatal risks in 4601 singletons and 884 twins conceived after fresh blastocyst transfers: a Nordic study from the CoNARTaS group. *Hum Reprod* **35**, 805–815 (2020).
17. Sazonova, A., Källen, K., Thurin-Kjellberg, A., Wennerholm, U.-B. & Bergh, C. Factors affecting obstetric outcome of singletons born after IVF. *Hum Reprod* **26**, 2878–2886 (2011).
18. Fernando, D., Halliday, J. L., Breheny, S. & Healy, D. L. Outcomes of singleton births after blastocyst versus nonblastocyst transfer in assisted reproductive technology. *Fertil Steril* **97**, 579–584 (2012).
19. Healy, D. L. *et al.* Prevalence and risk factors for obstetric haemorrhage in 6730 singleton births after assisted reproductive technology in Victoria Australia. *Hum Reprod* **25**, 265–274 (2010).
20. Ginström Ernstad, E. *et al.* Neonatal and maternal outcome after blastocyst transfer: a population-based registry study. *American Journal of Obstetrics and Gynecology* **214**, 378.e1-378.e10 (2016).
21. Opdahl, S. *et al.* Data Resource Profile: Committee of Nordic Assisted Reproductive Technology and Safety (CoNARTaS) cohort. *Int J Epidemiol* **49**, 365–366f (2020).
22. Rabe-Hesketh, S. & Skrondal, A. *Multilevel and longitudinal modeling using Stata*. (STATA press, 2008).
