# Supplementary material for: Risk of placenta previa in assisted reproductive technology: A Nordic population study with sibling analyses
Source: PLoS Med. 2025 Feb 3;22(2):e1004536. doi: 10.1371/journal.pmed.1004536 (PMC11835333; doi:10.1371/journal.pmed.1004536)
Supplement: Attachment — Submitted filename: Response_to_reviewers (2).docx [file pmed.1004536.s010.docx]

To the Editor

PLOS Medicine

**Manuscript ID PMEDICINE-D-24-02651R1: RESPONSE TO REVIEWERS AND EDITORS**

We thank the reviewers and Editors for their valuable comments and the opportunity to improve and resubmit our work. Our responses are outlined below. We have assigned numbers to the different points made by the reviewers and the Editor to be able to refer to specific points when there was overlap between the comments. We hope you will find all comments to be adequately addressed. In addition to the changes in the manuscript requested by the reviewers, we have made some small editions and corrections to make the content clearer. These changes are marked with Track Changes along with the requested changes but are not further commented in this letter.

Yours sincerely,

Eirik Landsverk

**********************************************************************************

Dear Dr Landsverk,

Many thanks for submitting your manuscript "Risk of placenta previa in assisted reproductive technology: A Nordic population study with sibling analyses" (PMEDICINE-D-24-02651R1) to PLOS Medicine. The paper has been reviewed by subject experts and a statistician; their comments are included below and can also be accessed here:

After discussing the paper with the editorial team and an academic editor with relevant expertise, I'm pleased to invite you to revise the paper in response to the reviewers' comments. We plan to send the revised paper to some or all of the original reviewers, and we cannot provide any guarantees at this stage regarding publication.

When you upload your revision, please include a point-by-point response that addresses all of the reviewer and editorial points, indicating the changes made in the manuscript and either an excerpt of the revised text or the location (eg: page and line number) where each change can be found. Please also be sure to check the general editorial comments at the end of this letter and include these in your point-by-point response. When you resubmit your paper, please include a clean version of the paper as the main article file and a version with changes tracked as a marked-up manuscript. It may also be helpful to check the guidelines for revised papers at <http://journals.plos.org/plosmedicine/s/revising-your-manuscript> for any that apply to your paper.

We ask that you submit your revision by Dec 19 2024 11:59PM. However, if this deadline is not feasible, please contact me by email, and we can discuss a suitable alternative.

Don't hesitate to contact me directly with any questions ([lgaynor@plos.org](mailto:lgaynor@plos.org)).

Best regards,

Louise

Louise Gaynor-Brook, MBBS PhD

Senior Editor

PLOS Medicine

[lgaynor@plos.org](mailto:lgaynor@plos.org)

-----------------------------------------------------------

Comments from the academic editor:

I agree that the fact the data goes back a long way is a significant limitation: this, and the potential impact of changing ART techniques, could be discussed in more depth. I am not convinced of the need for a country stratified analysis.

*We have included a discussion of these limitations, and we refer to our responses to comments 1.4 and 2.3 below where all the changes are presented.*

-----------------------------------------------------------

Comments from the reviewers:

Reviewer #1: In this paper, the authors conduct a retrospective registry study examining the relative odds of placenta previa in pregnancies using assisted retroductive technology (ART) vs. natural conception (NC). Major strengths of this study include the large sample size derived from a set of high quality national registries, which allows for the ability/power to examine specific sub-populations and sensitivity comparisons, and the use of a within-mother analysis in order to reduce heterogeneity due to mother-specific confounders. In general, we commend the authors on this well conducted study. A few suggestions are provided below.

1.1) I actually thought that Figure 2 regarding the nine potential combinations of first vs. second delivery modality deserved much more discussion than was provided in the manuscript. Some important patterns: we see lower predicted risk of placenta previa between the first delivery and second delivery when the first delivery is fresh-ET /except/ when the second delivery is also fresh-ET. Additionally, the magnitude of the increased predicted risk for first-delivery-NC pairs seems to vary greatly depending on the modality of the second delivery. These "differences in differences" so to speak are potentially of clinical interest, though they are only observational associations found in the data.

*We agree that figure 2 could receive more attention and have updated our results section accordingly:*

*Old: “Predicted risk of placenta previa tended to be higher after fresh-ET, both in the first and second delivery, irrespective of conception method in the preceding or succeeding delivery (Figure 2).”*

*New: ”The risk of placenta previa in the second delivery was highest after fresh-ET, intermediate after frozen-ET and lowest after NC when stratified according to conception method in the first delivery (Figure 2). Notably, the risk increased between first and second delivery for all combinations of conception method, except when fresh-ET was followed by frozen-ET or NC, where the risk was lower in the second delivery compared to the first.”*

*In addition, we have updated the discussion section of the manuscript:*

*“Although we cannot rule out confounding from non-stable maternal factors, we note that the risk of placenta previa was highest after fresh-ET regardless of delivery order among women who gave birth after different conception methods in their first two consecutive births.”*

1.2) Throughout the manuscript, there is occasional use of unsubstantiated causal language, which is not acceptable given the observational nature of the study (e.g., line 233-234: "ART increased the risk" as opposed to "ART was associated with increased risk beyond..."). One major area where we believe this language should definitely be changed is in line 71-72 ("assisted reproductive technology contributed to the increased risk"), which is a fully causal statement that was not supported by the analysis (the authors did not conduct a causal inference-informed approach). Despite the sibling design and the within-mother matching, and the comments on lines 288-290, such statements are still not appropriate. For instance, there still may remain potential unmeasured confounding due to time-varying factors between pregnancies. Thus, in the conclusion lines 319-320, it is also inappropriate to state that the higher observed risk is "attributed" to a combination of treatment and maternal factors (even if there is a biologically plausible explanation). To be most appropriate, we would prefer language such as "the highest risk of placenta previa after ART is associated with both...". For some more minor examples of problematic causal language, it may be more appropriate in lines 69-70 to state "The largest difference in risk was seen" as opposed to "The highest risk increase," as the latter (current) language implies a causal relationship.

*We have made changes throughout the manuscript to modify the use of causal language.*

1.3) Along these lines, it may be worth stating that the confounders referred to in lines 157-159 are /previously known confounders/ (with the implication being that there may always be unmeasured confounders, which will certainly be the case in large registry studies such as this one, e.g., environmental exposures, etc

*Old: “Available data on factors influencing the probability of ART conception and the risk of placenta previa (i.e., confounders) were included as categorical covariates; year of delivery, maternal age, parity and country.”*

*New: “Available data on factors known to influence the probability of ART conception and the risk of placenta previa were included as covariates in the regression models; year of delivery (categorical: 1988–1996, 1997–2001, 2002–2006, 2007–2011, 2012–2015), maternal age (categorical: 20–24, 25–29, 30–34, 35–39, 40–45), parity (categorical: 0, 1, 2, 3) and country.”*

1.4) Although the authors mention that a country-stratified analysis was not performed, it still may be of interest, perhaps due to potentially different medical practices or circumstances between (admittedly very similar) countries; even in a simple additionally sensitivity analysis that simply incorporated country as a categorical variable to be adjusted for, is there truly no evidence of difference across country lines? The inclusion of this variable is unlikely to affect inferential results if the relationship is truly null given the large sample size; it struck me as a strange modeling choice not to use data so immediately available and that may be potentially worth exploring.

*We have in fact included country as a categorical covariate in all analyses except those within mothers (where it will be constant). For the reasons that the reviewer pointed out, we would like to have done analyses of each country separately for the four subcategories of ART-conception (i.e. fresh-CT, frozen-CT, fresh-BT, frozen-BT), but unfortunately, we do not have enough data to do this for all countries, which is what we meant by these sentences in the discussion:*

*Old: “We had limited data on treatment details, especially culture duration, which was missing for a substantial proportion of ART-conceived pregnancies. Consequently, we did not perform analyses stratified by country, or by combinations of treatment and infertility factors.”*

*We have now revised this to make it clearer:*

*New: “We had limited data on ART treatment details, which was missing for all Finnish ART-pregnancies. Furthermore, cryopreservation and blastocyst transfers were uncommon in the first half of our study period. Consequently, there were not enough pregnancies after fresh-BT and frozen-BT to do country specific analyses. Similarly, we did not have sufficient statistical power to explore the risk placenta previa after fresh cleavage stage embryo transfer (fresh-CT), frozen-CT, fresh-BT and frozen-BT according to infertility factors.“*

*Our research group has previously reported that the incidence of placenta previa is slightly higher in Denmark and Finland compared to Norway and Sweden, but that the association between ART and NC appeared consistent across all countries [1].*

1.5) On a somewhat related note, I appreciated your discussion of the generalizability of the study; I would have preferred if you were even more explicit in mentioning that Nordic countries may not have populations which are comparable to those of other countries. For instance, you do discuss the difference in overall rate of placenta previa with respect to Ishihara et al., but it would have been stronger to explicitly mention this potential drawback (i.e., generalizing to other populations) as well.

*We agree and have made changes to the manuscript accordingly:*

*New: “Nevertheless, caution is needed when generalizing our findings to populations outside the Nordic countries, as no clear pattern between placenta previa, embryo culture duration and cryopreservation status was found in a large registry-based study from Japan (Ishihara et al.).”*

1.6) Finally, as a very small curiosity of mine, I wonder whether parity was included as a linear or categorical variable in your models when adjusting for them. Some additional clarity there would be appreciated.

*Parity was included as a categorical variable (0, 1, 2, 3). We agree that this should be clearer in the manuscript and have made changes accordingly (see point 1.3)*

In any case, a well-written, clear, and well-conducted study. It is likely to be of great interest to epidemiologists and practitioners.

*Thank you for this recognition.*

Reviewer #2: This is an excellent study performed by a highly experienced group of investigators, using a combined Nordic dataset that has been used previously. The study question was whether ART increases the risk for placenta previa. The authors evaluate this in a full population of ART and Naturally Conceived (NC) pregnancies as well as sequential pregnancies within the same woman. They analyze over 5 million pregnancies in 3 million mothers. They find that ART increases the rate of placenta previa and that specific ART techniques including fresh transfer (compared to frozen) and blastocyst transfer (compared to cleavage stage) showed the highest rates. In addition, specific infertility diagnoses were investigated and showed that endometriosis patients had a higher rate of placenta previa.

The data analysis in the paper is exhaustive. The tables and figures are appropriate, and the reference list appears comprehensive.

2.1) I had one question about the study population as described in Methods. It was not clear to me (perhaps I missed it), what was done with more than 2 pregnancies per woman. I assume that all pregnancies were included in the "Eligible sample". For the within woman comparisons, were just the first 2 pregnancies compared or was pregnancy 1 compared with pregnancy 2 and then pregnancy 2 compared with pregnancy 3? This should be clarified.

*We agree that this should be clarified and have made the following changes to the manuscript:*

*Old: “For population level estimates, we used random intercept models, whereas estimates within mothers were obtained using a fixed intercept model [41]. In the latter model, deliveries contribute to the final exposure-outcome estimate only if both the exposure and outcome differ between pregnancies by the same mother.”*

*New: “Population level associations were estimated in random intercept models using the full analysis samples, where each mother contributed 1-4 deliveries (multifetal pregnancy counting as one delivery). Within mother associations were estimated using a fixed intercept model, where each mother contributed 2-4 deliveries [41]. In the latter model, deliveries contribute to the final exposure-outcome estimate only if both the exposure and outcome differ between pregnancies by the same mother. As a sensitivity analysis, we restricted both models to only include the first two consecutive deliveries by each mother.”*

2.2) Also, was any calculation done to adjust for multiple pregnancies to the same woman in the overall analysis?

*Yes, we did. We agree that this should be clearer in the manuscript:*

*Old: “We estimated adjusted odds ratios (aORs) for placenta previa at the population level and within mothers using multilevel logistic regression with deliveries nested within mothers.”*

*New: “We estimated adjusted odds ratios (aORs) for placenta previa at the population level and within mothers using multilevel logistic regression with pregnancies nested within mothers. This hierarchical approach adjusts for clustering of pregnancies by the same mother.“*

2.3) I think that the authors should add the limitation that the study period spanned the years 1988-2015 which means it includes very old cases and that it does not include the most recent cases. While year was adjusted for, there have been many changes to ART protocols over the years that could make a difference.

*We agree that the relevance of the findings for today’s ART treatment might be questioned due to the lack of more recent data, and we have included the following argumentation:*

*New: “**ART treatment as well as the ART-treated populations continues to change, thus, there is a possibility that our findings of a higher risk of placenta previa after ART might not apply to women receiving treatment today. Although the newest data in this study were from 2015, we expect the findings to apply to contemporary treatment practices such as single embryo transfer and blastocyst culture. Fresh-BT now constitutes 18-21% of all Swedish embryo transfers, and frozen-BT has become far more prevalent than frozen-CT [2]. Indeed, a previous study on the CoNARTaS cohort showed a steadily increasing risk of placenta previa after ART compared to NC between 1988 and 2015, which might partly be explained by a shift from cleavage stage to blastocyst embryo transfer [1].”*

Reviewer #3: Congratulations on a very substantial body of work establishing that it is likely that presented previous is more common in IRT with variations dependent on the cause of infertility the staging of embryo transfer and particular interest presence of endometriosis. As much as I appreciate the statistical value of odds ratios, as a clinician the raw numbers are probably more relevant when counselling patients.

3.1) I would ask that the the final sentence in the conclusion is unnecessary. It perhaps should be From a clinician's perspective, the increased risks of placenta praevia, which is rarely a lifethreatening complication, are unlikely to change the chosen ART treatment approach. The primary goal of maximum pregnancy success will override these statistical findings.

*We agree that the last sentence could be more nuanced:*

*Old: “The risk of placenta previa should be balanced against risk of other complications when deciding on a fresh cycle attempt vs freezing of all embryos.”*

*New: “Although the risk of placenta previa is unlikely to be weighted heavily when choosing the type of ART treatment, it might be considered along with the risk of other and more severe pregnancy complications, especially if the alternative treatment types will not reduce the chances of achieving a pregnancy.”*

3.2) section rates but against a background of high rates after ART, the extra numbers will be relatively small .

*Unfortunately, we are not able to interpret this comment.*

References:

1. Petersen SH, Bergh C, Gissler M, Åsvold BO, Romundstad LB, Tiitinen A, et al. Time trends in placenta-mediated pregnancy complications after assisted reproductive technology in the Nordic countries. Am J Obstet Gynecol. 2020;223: 226.e1-226.e19. doi:10.1016/j.ajog.2020.02.030

2. Q-IVF. National reports from Q-IVF (Nationellt kvalitetsregister för assisterad befruktning). 2023 [cited 12 Dec 2024]. Available: https://www.medscinet.com/qivf/arsrapporter.aspx
