# Supplementary material for: Risk of placenta previa in assisted reproductive technology: A Nordic population study with sibling analyses
Source: PLoS Med. 2025 Feb 3;22(2):e1004536. doi: 10.1371/journal.pmed.1004536 (PMC11835333; doi:10.1371/journal.pmed.1004536)
Supplement: Attachment — Submitted filename: Landsverk_edits.docx [file pmed.1004536.s011.docx]

**Risk of placenta previa in assisted reproductive technology: A Nordic population study with sibling analyses**

Eirik Landsverk^1^*, Kjersti Westvik-Johari^1,2^, Ulla-Britt Wennerholm^3^, Christina Bergh^3^, Frederik Kyhl^4^, Anne Lærke Spangmose^4^, Ditte Vassard^4^, Anja Pinborg^4,5^, Kristiina Rönö^6^, Mika Gissler^7,8^, Sindre H. Petersen^1^, and Signe Opdahl^1^

^1^ Department of Public Health and Nursing, Faculty of Medicine and Health Science, Norwegian University of Science and Technology, Trondheim, Norway

^2^ Department of Fertility, Division of Obstetrics and Gynecology, St. Olavs Hospital, Trondheim University Hospital, Trondheim, Norway

^3^ Department of Obstetrics and Gynecology, Institute of Clinical Sciences, Sahlgrenska Academy, University of Gothenburg, Sahlgrenska University Hospital, Sweden

^4^ Fertility Clinic, Department of Gynaecology, Fertility and Obstetrics, Copenhagen University Hospital - Rigshospitalet, Denmark

^5^ Department of Clinical Medicine, University of Copenhagen, Copenhagen, Denmark

^6^ Department of Obstetrics and Gynecology, University of Helsinki and Helsinki University Hospital, Helsinki, Finland

^7^ Department of Data and Analytics, THL Finnish Institute for Health and Welfare, Helsinki, Finland

^8^ Department of Molecular Medicine and Surgery, Karolinska Institutet, Stockholm, Sweden

* Corresponding author

E-mail: [eirik.landsverk@ntnu.no](mailto:eirik.landsverk@ntnu.no) (EL) **Abstract**

BACKGROUND: A higher risk of placenta previa after assisted reproductive technology (ART) is well established. The underlying mechanisms are poorly understood, but may relate to embryo culture duration, cryopreservation, and cause of infertility. Within-mother analyses, where each woman is her own control (i.e., sibling design), help disentangle treatment contributions from maternal confounders that are stable between pregnancies. We aimed to investigate the risk of placenta previa in pregnancies achieved after ART according to embryo culture duration, cryopreservation, and infertility factors while accounting for stable maternal factors using within-mother analyses.

METHODS and Findings: We used linked nationwide registry data from Denmark (1994–2014), Finland (1990–2014), Norway (1988–2015), and Sweden (1988–2015). All women who gave their first birth during the study period at age 20 years or older, were eligible and contributed with ≤4 deliveries (singleton or multifetal) at 22–44 gestational weeks before age 45 years. We used multilevel logistic regression to compare risk of placenta previa after ART (n=139,694 deliveries) vs natural conception (n=5,614,512 deliveries), both at the population level and within mothers, adjusting for year of delivery, maternal age, parity, and country. We categorized ART according to culture duration, embryo cryopreservation and infertility factors. Population level risk of placenta previa was higher for ART vs natural conception (odds ratio [OR], 4.16; 95% confidence interval [CI], 3.96–4.37). Controlling for stable maternal factors, the association attenuated, but risk remained higher for ART vs natural conception (OR within mothers, 2.64; 95% CI, 2.31–3.02). Compared to naturally conceived, a larger difference in risk was seen for pregnancies from fresh embryos than for pregnancies from frozen embryos. Further categorization by culture duration showed the largest risk difference after fresh blastocyst transfer, and the smallest after frozen cleavage stage embryo transfer, which persisted in sensitivity analyses (including restriction to singletons). When stratified according to infertility factors at the population level, women with endometriosis conceiving by ART had the highest risk of placenta previa (OR, 9.35; 95% CI, 8.50–10.29), whereas women with polycystic ovary syndrome (PCOS) conceiving by ART had the lowest risk (OR, 1.52; 95% CI, 1.12–2.09), compared to natural conception. Within mothers, we found a higher risk of placenta previa after ART compared to natural conception for women with endometriosis (OR, 2.08; 95% CI, 1.50–2.90), but not for women with PCOS (OR, 0.88; 95% CI, 0.41–1.89 [unadjusted due to sparse data]). However, within-mother analyses are restricted to multiparous women with deliveries after different conception methods. Therefore, findings from these analyses might not generalize to all women undergoing ART.

CONCLUSIONS: The risk of placenta previa in pregnancies conceived by ART differed by embryo culture duration, cryopreservation, and underlying infertility. The highest risk was seen after fresh embryo transfer, and especially fresh blastocyst transfer. Women with endometriosis had a higher risk than women with other infertility factors, and within mothers, their risk was higher after ART than after natural conception. Identifying the responsible mechanisms might provide opportunities for prevention.

# **Author summary**

Why Was This Study Done?

- Studies indicate risk of placenta previa in assisted reproductive technology (ART) differs by type of ART method and underlying infertility.
- We aimed to increase our understanding of these factors’ contributions by improved confounding control using within-mother (sibling) analyses.

What Did the Researchers Do and Find?

- Using each mother as her own control, we found the highest risk of placenta previa after fresh blastocyst transfer.
- In addition, we found a markedly higher risk among women with endometriosis who conceived after assisted reproductive technology, which remained doubled compared to natural conception after controlling for maternal factors through within-mother analyses.

What Do These Findings Mean?

- This study strengthens evidence that treatment and maternal factors may both contribute to risk of placenta previa in assisted reproductive technology.
- Further insights into the mechanisms responsible might lead to development of safer ART methods.
- Although within-mother analyses are less prone to confounding from stable maternal factors, the findings of these analyses require that women have several births, hence, results might not apply to all women using ART.

# **Introduction**

The use of assisted reproductive technology (ART) is increasing worldwide [1]. This can be attributed to increasing success rates and availability of ART, and postponement of parenthood to an age with lower fecundity [2,3]. Compared to natural conception (NC), ART is associated with higher risk of pregnancy complications such as hypertensive disorder of pregnancy, gestational diabetes mellitus and placenta previa [4–6], as well as adverse perinatal outcomes including small for gestational age, preterm birth and perinatal death [7].

In Europe, more than 200,000 children are born after ART each year (constituting 3% of the total birth cohorts), and transfer of single embryos has greatly reduced multifetal gestations and hence perinatal complications in ART-conceived pregnancies [3,8,9]. In contrast, the risk of placenta previa after ART has increased over time in the Nordic countries [10], which is concerning as placenta previa is associated with complications for the mother and child [11–17].

Previous studies have linked both fresh embryo transfer (fresh-ET) and blastocyst transfer to increased risk of placenta previa [18–22], and the combination of these two; fresh blastocyst transfer (fresh-BT), has been linked to a particularly high risk in some studies [23–26], but not in others [27,28]. Differences in underlying infertility might explain some of the discrepancies between previous studies [18,29,30]. Separating the contributions from underlying infertility and ART may be possible with a within-mother design, i.e., sibling design [31,32].

In a large study population, we investigated the risk of placenta previa according to embryo culture duration, cryopreservation, and infertility factors, accounting for maternal factors using within-mother analyses.

# **Materials and Methods**

We used data from the Committee of Nordic Assisted Reproductive Technology and Safety (CoNARTaS) cohort, which comprises all deliveries in the Medical Birth Registries (MBRs) in Denmark, Finland, Norway and Sweden since the start of national ART registration. The MBRs collect information on all deliveries in each country. The mothers’ unique national identity number was used for individual data linkage between the MBRs and other national health registries [33].

## **Data sources and variables**

The exposure was conception by ART, defined as fertilization outside the female body, i.e. fertilization with in vitro fertilization (IVF) or intracytoplasmic sperm injection [34]. ART-conception was identified through ART registries in Denmark and Sweden, and MBRs in Norway and Finland [33]. Pregnancies after ovulation induction and insemination were categorized as naturally conceived together with pregnancies not registered as ART-conceived. Danish, Norwegian and Swedish ART data could be further categorized as cleavage stage embryo transfer (culture duration 2–3 days) or blastocyst transfer (culture duration 5–6 days), and as fresh-ET or frozen thawed embryo transfer (frozen-ET).

The outcome was placenta previa, identified from the MBRs in Finland, Norway and Sweden, and the National Patient Registries of Denmark and Finland (S1 Table). Using these registries, we could not differentiate between total, partial or marginal placenta previa, or low-lying placenta (<2 cm from the internal os). We only included diagnoses during the third trimester or within one month before delivery from the National Patient Registries because most low-lying placentas in the second trimester resolve in the third trimester [35]. In the Nordic countries, placenta previa is usually discovered at second trimester routine ultrasonography, and follow-up is normally performed in week 32–34 to evaluate whether it has resolved [36–38].

Infertility factors (i.e., diagnoses associated with infertility) were identified using the National Patient Registries of each country, supplemented with information from the national IVF registry in Denmark, and the Medical Birth Registry of Norway (S2 and S3 Tables). We assigned each woman to only one of these factors during the study period. Factors more strongly associated with the risk of placenta previa [18,29,30], and factors with low prevalence, were given priority in this order: uterine factors, endometriosis, polycystic ovary syndrome (PCOS), male-factor infertility only, and other infertility factors (including unspecified female infertility and unexplained infertility).

## **Study population**

The study period was defined as 1994–2014 in Denmark, 1990–2014 in Finland, and 1988–2015 in Norway and Sweden. Only deliveries by women whose first delivery occurred during the study period at age ≥20 years were defined as eligible (Fig 1). This was done to ensure sufficient overlap in year of delivery, maternal age, and parity between ART and NC [39]. For these women, up to the first four deliveries were included (parity 0–3), excluding deliveries at maternal age >45 years. In addition, deliveries with extreme (<22 or >44 weeks) or missing gestational age were excluded. We included multifetal pregnancies because this does not seem to be a clear risk factor of placenta previa [9,10,30,40]. ART-conceived pregnancies after gamete donation and/or with preimplantation genetic testing were also included. **Sample 1** included 5,754,206 deliveries in all four countries combined. Among these, 139,694 were from ART-conceived pregnancies, and 42,792 women gave birth after both ART and NC during the study period (Fig 1).

**Fig 1. Flow chart of study population showing eligibility, exclusion criteria and numbers, and number of exposed and unexposed in the three analyses samples.** Abbreviations: ART, assisted reproductive technology; fresh-ET, fresh embryo transfer; frozen-ET, frozen embryo transfer; fresh-CT, fresh cleavage stage embryo transfer; fresh-BT, fresh blastocyst transfer; frozen-CT, frozen cleavage stage embryo transfer; frozen-BT, frozen blastocyst transfer.

In **sample 2**, ART-conceived pregnancies with missing cryopreservation status were excluded. To create **sample 3**, ART-conceived pregnancies with missing culture duration were excluded, leaving 94,631 deliveries after ART for analyses on combinations of culture duration and cryopreservation (Fig 1). We also excluded pregnancies after NC from Finland in samples 2 and 3 as there was no information on culture duration or cryopreservation among ART-conceived pregnancies in Finland.

## **Statistical analyses**

We estimated adjusted odds ratios (aORs) for placenta previa at the population level and within mothers using multilevel logistic regression with deliveries nested within mothers. This hierarchical approach adjusts for clustering of pregnancies by the same mother. Population level associations were estimated in random intercept models using the full analysis samples, where each mother contributed 1-4 deliveries (multifetal pregnancy counting as one delivery). Within mother associations were estimated using a fixed intercept model [41], where each mother contributed 2-4 deliveries. In the latter model, deliveries contribute to the final exposure-outcome estimate only if both the exposure and outcome differ between pregnancies by the same mother. As a sensitivity analysis, we restricted both models to only include the first two consecutive deliveries by each mother. Available data on factors known to influence the probability of ART conception and the risk of placenta previa were included as covariates in the regression models; year of delivery (categorical: 1988–1996, 1997–2001, 2002–2006, 2007–2011, 2012–2015), maternal age (categorical: 20–24, 25–29, 30–34, 35–39, 40–45), parity (categorical: 0, 1, 2, 3) and country.

We used all NC pregnancies as the reference in population level analyses of infertility factors. Within mothers, we performed separate analyses for each infertility factor, restricting analyses to women assigned to that factor.

In sample 2, we examined whether risk of placenta previa within mothers was driven by certain combinations of parity and conception method with ART categorized as either fresh-ET or frozen-ET. Specifically, we repeated the multilevel logistic regression at the population level with an interaction term between parity and the resulting nine combinations of conception methods for the mothers’ first and second delivery and obtained predicted risks of placenta previa using postestimation commands. We also did a series of other sensitivity analyses to test the robustness of the results, some of which are described in more detail in S1 Text.

Finally, to assess the clinical severity of placenta previa, we compared other pregnancy outcomes (gestational age, birthweight and caesarean section) between deliveries with and without placenta previa according to conception method and plurality.

We used Stata 18 (Statacorp LLC, TX, USA) for all statistical analyses.

This study is reported as per the Reporting of Studies Conducted using Observational Routinely-Collected Data (RECORD) guideline (S1 Checklist). All analyses were planned a priory as outlined in S1 Study Protocol.

## **Ethics**

In Norway, the study was approved by the Regional Committees for Medical and Health Research Ethics (REK 2010/1909). In Sweden, the study was approved by the Ethical committee in Gothenburg (Dnr 214-12, T422-12, T516-15, T233-16, T300-17, T1144-17, T121-18). In Denmark and Finland, ethical approval is not required for research only based on registry data.

# **Results**

Background characteristics differed between pregnancies after ART and NC (Table 1). The number of deliveries after ART increased over the study period. Women who conceived by ART were on average four years older, had lower parity and less frequently smoked during pregnancy, compared to naturally conceiving women. In addition, twin pregnancies were more common after ART than NC (14.0% vs 1.3%).

| **Table 1.** Description of the study population in sample 1. | | |
| --- | --- | --- |
|  | ART | NC |
| Total number of pregnancies, n | 139,694 | 5,614,512 |
| Country, n (%) |  |  |
| Denmark | 35,365 (25.3) | 1,001,886 (17.8) |
| Finland | 23,835 (17.1) | 1,111,804 (19.8) |
| Norway | 26,805 (19.2) | 1,217,113 (21.7) |
| Sweden | 53,689 (38.4) | 2,283,709 (40.7) |
| Year of birth, n (%) |  |  |
| 1988-1996 | 11,624 (8.3) | 1,295,414 (23.1) |
| 1997-2001 | 22,371 (16.0) | 1,046,979 (18.7) |
| 2002-2006 | 30,251 (21.7) | 1,165,294 (20.8) |
| 2007-2011 | 41,727 (29.9) | 1,234,664 (22.0) |
| 2012-2015 | 33,721 (24.1) | 872,161 (15.5) |
| Mean maternal age (SD) | 33.7 (4.2) | 29.6 (4.8) |
| Parity, n (%) |  |  |
| 0 | 100,857 (72.2) | 2,862,509 (51.0) |
| 1 | 33,881 (24.3) | 1,998,172 (35.6) |
| 2 | 4251 (3.0) | 620,801 (11.1) |
| 3 | 705 (0.5) | 133,030 (2.4) |
| Smoking^a^, n (%) |  |  |
| Yes | 7198 (5.3) | 610,606 (11.8) |
| No | 120,830 (89.2) | 4,280,132 (82.5) |
| Missing^b^ | 7362 (5.4) | 299,460 (5.8) |
| Maternal BMI^c^ (kg/m^2^), mean (SD) | 24.2 (4.1) | 24.2 (4.5) |
| Missing^b^, n (%) | 14,210 (13.4) | 538,828 (14.5) |
| Infertility factors |  |  |
| Uterine factor^d^ | 1517 (1.1) | NA |
| Endometriosis^e^ | 16,205 (11.6) | NA |
| Polycystic ovary syndrome^f^ | 7051 (5.1) | NA |
| Male factors only^g^ | 10,222 (7.3) | NA |
| Other infertility factors^h^ | 97,401 (70.0) | NA |
| Missing^i^ | 7298 (5.2) | NA |
| Plurality, n (%) |  |  |
| Singleton | 119,591 (85.6) | 5,540,830 (98.7) |
| Twin | 19,513 (14.0) | 72,504 (1.3) |
| Triplet or higher | 590 (0.4) | 1178 (0.0) |
| Abbreviations: ART, assisted reproductive technology; NC, natural conception, NA, not applicable. | | |
| ^a^Smoking was recorded from 1999 in Norway, and prior to our study period in Denmark, Finland and Sweden. | | |
| ^b^Missing during registration period. | | |
| ^c^BMI was recorded from 2007 in Norway, 2004 in Denmark and Finland, and prior to our study period in Sweden. | | |
| ^d^Pregnancies by women once diagnosed with female infertility due to uterine factors. | | |
| ^e^Pregnancies by women once diagnosed with endometriosis without any diagnosis of female infertility due to uterine factors. | | |
| ^f^Pregnancies by women once diagnosed with polycystic ovary syndrome, without any diagnosis of female infertility due to uterine factors nor endometriosis. | | |
| ^g^Pregnancies by women once diagnosed with infertility due to male factors, without any diagnosis of infertility due to female factors. | | |
| ^h^Pregnancies by women with other infertility factors than uterine factors, endometriosis, PCOS and male factors only. This category also includes pregnancies by women with unspecified female infertility and unexplained infertility. | | |
| ^i^Pregnancies by women missing any diagnosis code associated with infertility. | | |

For ART vs NC at the population level, aOR of placenta previa was 4.16 (95% confidence interval [CI], 3.96–4.37). Comparisons within mothers who delivered after both ART and NC, also showed a clearly higher risk after ART, although the point estimate was lower than at the population level (aOR, 2.64; 95% CI, 2.31–3.02). At the population level, risk was higher after fresh-ET (aOR, 4.69; 95% CI, 4.41–4.98) than after frozen-ET (aOR, 2.71; 95% CI, 2.35–3.13), using NC as reference. When further categorizing fresh-ET and frozen-ET according to culture duration, population level risk was higher for all treatment combinations compared to NC (Table 2), with the highest risk after fresh-BT (aOR, 11.51; 95% CI, 9.75–13.57). Within mothers, risk estimates attenuated to some extent, but remained higher for all treatment combinations except frozen cleavage stage embryo transfer (frozen-CT, Table 2).

| **Table 2.** Observed risk and adjusted odds ratio of placenta previa at the population level and within mothers according to conception method and sample | | | | | | |
| --- | --- | --- | --- | --- | --- | --- |
|  | Population level | | | Within mothers | | |
|  | N | Observed risk | Adjusted odds ratio^a^ (95% CI) | N^b^ | Observed risk^c^ | Adjusted odds ratio^d^ (95% CI) |
| Sample 1 |  |  |  |  |  |  |
| NC | 5,614,512 | 3.1/1000 | 1 (ref) | 51,521 | 7.3/1000 | 1 (ref) |
| ART | 139,694 | 17.2/1000 | 4.16 (3.96 to 4.37) | 44,843 | 17.8/1000 | 2.64 (2.31 to 3.02) |
| Sample 2 |  |  |  |  |  |  |
| NC | 4,502,708 | 2.8/1000 | 1 (ref) | 39,237 | 6.4/1000 | 1 (ref) |
| Fresh-ET | 93,078 | 17.4/1000 | 4.69 (4.41 to 4.98) | 34,810 | 18.3/1000 | 3.31 (2.81 to 3.91) |
| Frozen-ET | 19,851 | 11.1/1000 | 2.71 (2.35 to 3.13) | 10,724 | 10.4/1000 | 1.59 (1.19 to 2.13) |
| Sample 3 |  |  |  |  |  |  |
| NC | 4,502,708 | 2.8/1000 | 1 (ref) | 32,182 | 6.6/1000 | 1 (ref) |
| Fresh-CT | 72,878 | 16.5/1000 | 4.24 (3.96 to 4.54) | 27,497 | 17.3/1000 | 3.07 (2.55 to 3.69) |
| Fresh-BT | 4882 | 41.0/1000 | 11.51 (9.75 to 13.57) | 2401 | 40.0/1000 | 7.80 (4.59 to 13.27) |
| Frozen-CT | 12,971 | 9.7/1000 | 2.33 (1.93 to 2.80) | 7303 | 7.9/1000 | 1.20 (0.83 to 1.75) |
| Frozen-BT | 3900 | 15.6/1000 | 4.04 (3.07 to 5.31) | 1768 | 19.8/1000 | 3.13 (1.71 to 5.73) |
| Abbreviations: CI, confidence interval; NC, natural conception; ART, assisted reproductive technology; fresh-ET, fresh embryo transfer; frozen-ET, frozen embryo transfer; fresh-CT, fresh cleavage stage embryo transfer; fresh-BT, fresh blastocyst transfer; frozen-CT, frozen cleavage stage embryo transfer; frozen-BT, frozen blastocyst transfer. | | | | | | |
| ^a^Adjusted for year of delivery (categorical: 1988–1996, 1997–2001, 2002–2006, 2007–2011, 2012–2015), maternal age (categorical: 20–24, 25–29, 30–34, 35–39, 40–45), parity (categorical: 0, 1, 2, 3) and country. | | | | | | |
| ^b^Total number of pregnancies from mothers with at least two different conception methods. | | | | | | |
| ^c^Observed risk in pregnancies from mothers with at least two different conception methods. | | | | | | |
| ^d^Adjusted for year of delivery, maternal age and parity (country is constant within mothers and therefore not included as a covariate in these analyses). Estimated by logistic regression (fixed intercept model) on all pregnancies in each sample. | | | | | | |

At the population level, ART-conception was associated with higher risk of placenta previa compared to NC for all infertility factors (Table 3), ranging from highest among women with endometriosis (aOR, 9.35; 95% CI, 8.50–10.29) to lowest among women with PCOS (aOR, 1.52; 95% CI, 1.12–2.09). Within mothers, the associations attenuated compared to the population level, but risk remained higher in ART vs NC for all infertility factors (Table 3), except for PCOS (odds ratio, 0.88; 95% CI, 0.41–1.89 [unadjusted due to sparse data]). For women with endometriosis, aOR was 2.08 (95% CI, 1.50–2.90) for ART vs NC within mothers. Giving endometriosis and PCOS first priority in the hierarchy of infertility factors, we found similar results both for endometriosis (population level aOR, 9.31; 95% CI, 8.47–10.24; aOR within mothers, 2.10; 95% CI, 1.51–2.92), and for PCOS (population level aOR, 1.89; 95% CI, 1.44–2.48; OR within mothers, 0.82; 95% CI, 0.41–1.65 [unadjusted due to sparse data]).

| **Table 3.** Observed risk and adjusted odds ratio of placenta previa at the population level and within mothers according to conception method and infertility factor in sample 1 | | | | | | |
| --- | --- | --- | --- | --- | --- | --- |
|  | Population level^a^ | | | Within mothers^b^ | | |
|  | N | Observed risk | Adjusted odds ratio^c^ (95% CI) | N^d^ | Observed risk^e^ | Adjusted odds ratio^f^ (95% CI) |
| Uterine factors^g^ |  |  |  |  |  |  |
| NC | 5,614,512 | 3.1/1000 | 1 (ref) | 472 | 6.4/1000 | 1 (ref) |
| ART | 1517 | 18.5/1000 | 3.76 (2.51 to 5.65) | 430 | 20.9/1000 | 3.06 (0.82 to 11.41)^h^ |
| Endometriosis^i^ |  |  |  |  |  |  |
| NC | 5,614,512 | 3.1/1000 | 1 (ref) | 5481 | 15.5/1000 | 1 (ref) |
| ART | 16,205 | 37.3/1000 | 9.35 (8.50 to 10.29) | 4983 | 30.7/1000 | 2.08 (1.50 to 2.90) |
| PCOS^j^ |  |  |  |  |  |  |
| NC | 5,614,512 | 3.1/1000 | 1 (ref) | 2434 | 6.6/1000 | 1 (ref) |
| ART | 7051 | 6.1/1000 | 1.53 (1.12 to 2.09) | 2159 | 5.6/1000 | 0.88 (0.41 to 1.89)^h^ |
| Male factors only^k^ |  |  |  |  |  |  |
| NC | 5,614,512 | 3.1/1000 | 1 (ref) | 3532 | 4.8/1000 | 1 (ref) |
| ART | 10,222 | 11.3/1000 | 3.04 (2.50 to 3.70) | 3110 | 13.5/1000 | 2.91 (1.64 to 5.17)^h^ |
| Other infertility factors^l^ |  |  |  |  |  |  |
| NC | 5,614,512 | 3.1/1000 | 1 (ref) | 39,723 | 6.4/1000 | 1 (ref) |
| ART | 104,699 | 15.4/1000 | 3.49 (3.30 to 3.69) | 34,377 | 17.0/1000 | 2.76 (2.31 to 3.29) |
| Abbreviations: CI, confidence interval; NC, natural conception; ART, assisted reproductive technology; PCOS, polycystic ovary syndrome. | | | | | | |
| ^a^At the population level, infertility factor was only defined for pregnancies after ART. | | | | | | |
| ^b^Within mothers, infertility factor was only defined for women with an ART conception during the study period. | | | | | | |
| ^c^Adjusted for year of delivery (categorical: 1988–1996, 1997–2001, 2002–2006, 2007–2011, 2012–2015), maternal age (categorical: 20–24, 25–29, 30–34, 35–39, 40–45), parity (categorical: 0, 1, 2, 3) and country. | | | | | | |
| ^d^Total number of pregnancies from mothers with pregnancies after both NC and ART during the study period. | | | | | | |
| ^e^Observed risk in pregnancies from mothers with pregnancies after both NC and ART during the study period. | | | | | | |
| ^f^Adjusted for year of delivery (categorical: 1988–1996, 1997–2001, 2002–2006, 2007–2011, 2012–2015), maternal age (categorical: 20–24, 25–29, 30–34, 35–39, 40–45), and parity (categorical: 0, 1, 2, 3). Country is constant within mothers and therefore not included as a covariate in these analyses. Estimated by logistic regression (fixed intercept model) on all pregnancies in each category of infertility factor. | | | | | | |
| ^g^Pregnancies by women once diagnosed with female infertility due to uterine factors. | | | | | | |
| ^h^The odds ratios comparing ART vs NC within mothers in women with uterine factors, PCOS and male factors only are unadjusted due to sparse data. | | | | | | |
| ^i^Pregnancies by women once diagnosed with endometriosis without any diagnosis of female infertility due to uterine factors. | | | | | | |
| ^j^Pregnancies by women once diagnosed with polycystic ovary syndrome, without any diagnosis of female infertility due to uterine factors nor endometriosis. | | | | | | |
| ^k^Pregnancies by women once diagnosed with infertility due to male factors, without any diagnosis of infertility due to female factors. | | | | | | |
| ^l^Pregnancies by women with other infertility factors than uterine factors, endometriosis, PCOS, and male factors only. This category also includes pregnancies by women with unspecified female infertility, unexplained infertility, and missing any diagnosis code associated with infertility. | | | | | | |

The risk of placenta previa in the second delivery was highest after fresh-ET, intermediate after frozen-ET and lowest after NC when stratified according to conception method in the first delivery (Fig 2). Notably, the risk increased between first and second delivery for all combinations of conception method, except when fresh-ET was followed by frozen-ET or NC, where the risk was lower in the second delivery compared to the first (Fig 2).

**Fig 2. Predicted risk of placenta previa in the two first deliveries according conception methods in sample 2.** Abbreviations: Frozen-ET, frozen embryo transfer; fresh-ET, fresh embryo transfer; NC, natural conception. We calculated predicted risk of placenta previa using multilevel logistic regression at the population level and marginal standardization (“margins” command in Stata [StataCorp, College Station, TX]). We included the following covariates in the regression model: parity (0, 1), conception methods for the mothers’ first and second delivery (categorical: NC – NC, NC – fresh-ET, NC – frozen-ET, fresh-ET – NC, fresh-ET – fresh-ET, fresh-ET – frozen-ET, frozen-ET – NC, frozen-ET – fresh-ET, frozen-ET – frozen-ET), year of delivery (categorical: 1988–1996, 1997–2001, 2002–2006, 2007–2011, 2012–2015), maternal age (categorical: 20–24, 25–29, 30–34, 35–39, 40–45) and country. We added an interaction term between parity and conception methods for the mothers’ first and second delivery to allow the slope of the lines to differ.

All sensitivity analyses were consistent with the main results. When stratified on characteristics of the first pregnancy (conception method and caesarean section/placenta previa), risk of placenta previa in the second pregnancy was consistently higher in ART-conceived pregnancies compared to NC (S4 and S5 Tables). Associations between embryo culture duration, cryopreservation, and placenta previa were also consistent across sensitivity analyses (Fig 3). Although results for fresh-BT and frozen blastocyst transfer (frozen-BT) attenuated somewhat within mothers when restricting ART-conceived pregnancies to fertilization with IVF (i.e., without intracytoplasmic sperm injection), risk still tended to be higher after fresh-BT than after other combinations of culture duration and cryopreservation status in this subpopulation (Fig 3).

**Fig 3. Adjusted odds ratio of placenta previa after (A) fresh-CT, (B) fresh-BT, (C) frozen-CT and (D) frozen-BT compared to NC in sample 3, at the population level and within mothers.** Abbreviations: Fresh-CT, fresh cleavage stage embryo transfer; NC, natural conception; fresh-BT, fresh blastocyst transfer; frozen-CT, frozen cleavage stage embryo transfer; frozen-BT, frozen blastocyst transfer. Adjusted for year of delivery (categorical: 1988–1996, 1997–2001, 2002–2006, 2007–2011, 2012–2015), maternal age (categorical: 20–24, 25–29, 30–34, 35–39, 40–45), parity (categorical: 0, 1, 2, 3) and country (only included in analyses at the population level because it is constant within mothers). Analyses in “Smoking” are additionally adjusted for smoking status in the subpopulation where this information was available. In “C-section” the outcome of placenta previa is restricted to pregnancies delivered by caesarean section, whereas “Full siblings”, “First two”, “<3 years”, “SET”, “IVF” and “Singletons” refer to restriction of analyses to full siblings (i.e., same mother and father), each mothers’ first two consecutive deliveries, only consecutive deliveries less than three years apart, single embryo transfer (using all deliveries after NC as reference), in vitro fertilization (using all deliveries after NC as reference), and singleton pregnancies, respectively.

ART-conceived singleton pregnancies with vs without placenta previa had shorter mean gestational age (259.0 vs 276.9 days), higher proportions of preterm birth (36.3% vs 7.5%) and lower mean birthweight (2986 vs 3447 g), but similar mean birthweight z-scores and proportions of small and large for gestational age. A similar pattern of lower birthweight and gestational age was observed in naturally conceived pregnancies and ART-conceived twin pregnancies complicated by placenta previa (S6 Table).

# **Discussion**

The increased risk of placenta previa in ART-conceived pregnancies differed by embryo culture duration and cryopreservation, both at the population level and within mothers, with a higher risk after fresh-ET, and especially fresh-BT. Estimates attenuated from population level to within mothers, suggesting stable maternal factors contribute to the increased risk. This was further supported by large differences in risk according to infertility factors, where women with ART-conception and endometriosis had the highest risk of placenta previa. Their risk after ART-conception remained higher in within-mother analyses, suggesting that their higher risk after ART could not be fully explained by stable maternal factors.

In line with our results, Spangmose et al., Korosec et al., Volodarsky-Perel et al., and Cirillo et al. reported risks of 4.1%, 4.0%, 3.5% and 4.3%, respectively, of placenta previa after fresh-BT, from a total of 4601, 802, 425 and 202 pregnancies, respectively [23–26]. The study by Spangmose et al. is a previous study on the CoNARTaS cohort. We shed new light on those findings by providing better control for confounding from stable maternal factors, and by improving our understanding of the contribution from different infertility factors.

A nationwide Japanese study of single embryo transfers (n=48,158) showed no clear differences across combinations of culture duration and cryopreservation but had an appreciably lower risk of placenta previa after ART-conception overall (0.7–0.9%) than in our study [27].

Endometriosis was strongly associated with placenta previa in ART-conceived pregnancies, consistent with previous comparisons of ART-conceiving women with endometriosis to either ART-conceiving women without endometriosis [18,30,41–43], or women who conceived naturally with or without endometriosis [29,42]. We elaborate these observations by using within-mother analyses to disentangle the contribution of ART from that of endometriosis. Our results indicate that ART is associated with a doubled odds of placenta previa in women with endometriosis, from an already higher baseline risk than among women with other infertility factors.

In ART, PCOS was associated with lower risk of placenta previa than other infertility factors. This is consistent with previous studies comparing women using ART for ovulation disorders to women with NC [29], and to women without ovulation disorders using ART [18,30]. In contrast to previous studies, we studied PCOS instead of ovulation disorders, because the latter also includes poor ovarian reserve due to advanced maternal age, which makes interpretation more challenging.

Several mechanisms for a higher risk of placenta previa after ART-conception have been suggested. One hypothesis is that the position of the catheter during embryo transfer might play a role [44], supported by observations that transfer site corresponds to implantation site in 80% of transfers [45]. Another hypothesis is that the catheter sometimes causes uterine contractions when passing through the cervix, which may displace the embryo [46]. Enhanced uterine peristalsis has also been hypothesized to play a role in the higher risk of previa in women with endometriosis [47].

Fresh-ET is associated with higher levels of estradiol and progesterone at the time of embryo transfer compared to frozen-ET, as a result of ovarian stimulation [48,49]. Increased levels of estradiol have been proposed to lower the threshold for uterine contractions in response to mechanical stimuli, which could explain the higher risk of placenta previa after fresh-ET [46]. However, a subsequent study found no association between peak estradiol during ovarian stimulation and subsequent risk of placenta previa after fresh-ET [47]. Further, a premature rise in progesterone levels has been shown to induce premature maturation of the endometrium [49]. It seems conceivable that a longer secretory phase might make the endometrium in the lower segment of the uterus more receptive to embryo implantation in fresh-ET compared to frozen-ET and NC. This effect may be enhanced in fresh-BT as extended culture duration results in prolonged maturation of the endometrium before embryo transfer. Indeed, thicker endometrium is associated with higher risk of placenta previa after fresh-ET [47,50,51], but not after frozen-ET [52,53]. A greater number of retrieved oocytes has been associated with higher progesterone levels [54]. We had no information on progesterone levels during ovarian stimulation, but a study of deliveries after fresh-ET in Sweden between 2002 and 2015 found that more oocytes retrieved was associated with higher risk of placenta previa in singleton pregnancies [55].

Future studies should explore the association between fresh-ET, premature rise in progesterone levels and subsequent risk of placenta previa. In addition, analyses of the risk of placenta previa according to embryo culture duration and cryopreservation in women with endometriosis, and women with other infertility factors, is warranted.

## **Strengths and limitations**

A key strength of this study is the within-mother analyses where each woman serves as her own control [56]. In a Norwegian study population that partly overlaps with ours, Romundstad et al. also found higher risk of placenta previa after ART compared to NC using within-mother analyses [44]. We assume such analyses are less prone to confounding from stable maternal factors such as cause of infertility and genetic predisposition for placenta previa. Although we cannot rule out confounding from non-stable maternal factors, we note that the risk of placenta previa was highest after fresh-ET regardless of delivery order among women who gave birth after different conception methods in their first two consecutive births. Another limitation of within-mother analyses is the restriction to multiparous women, who might not be representative of all women using ART.

We had limited data on ART treatment details, which was missing for all Finnish ART-pregnancies. Furthermore, cryopreservation and blastocyst transfers were uncommon in the first half of our study period [23,33]. Consequently, there were not enough pregnancies after fresh-BT and frozen-BT to do country specific analyses. Similarly, we did not have sufficient statistical power to explore the risk placenta previa after fresh cleavage stage embryo transfer (fresh-CT), frozen-CT, fresh-BT and frozen-BT according to infertility factors. While we cannot rule out information bias from differences in data sources and registration practice of placenta previa, we expect this to be non-differential with respect to conception method. Furthermore, we assume negligible information bias from defining pregnancies after ovulation induction and insemination as natural conceptions, because these pregnancies are very few compared to the remaining background population. Moreover, beyond restricting the diagnosis of placenta previa to deliveries with caesarean section in sensitivity analysis, we could not separate a total, partial or marginal placenta previa from a low-lying placenta. However, low-lying placentas also appear clinically relevant [57,58], and their management are similar to placenta previa [59].

The higher risk of placenta previa in women with endometriosis in our study might not reflect the risk associated with endometriosis in general, since we restricted diagnosis of endometriosis to women with delivery after ART. Of note, an ICD-code of endometriosis in the Swedish National Patient Registry had a positive predictive value of 97.8% compared to a diagnosis in medical records [60]. Furthermore, our assignment of women to specific infertility factor groups will not always reflect the main cause of infertility, which can also be difficult to ascertain clinically [61]. We had no data on the severity or diagnostic basis of endometriosis, both of which may be associated with the risk of placenta previa [62,63].

A strength of this study was the consistency of results across a series of sensitivity analyses. The population-based design with inclusion of both singleton and multifetal pregnancies is expected to better capture the total effect of ART compared to previous studies [21–27]. ART treatment as well as the ART-treated populations continues to change, thus, there is a possibility that our findings of a higher risk of placenta previa after ART might not apply to women receiving treatment today. Although the newest data in this study were from 2015, we expect the findings to apply to contemporary treatment practices such as single embryo transfer and blastocyst culture. Fresh-BT now constitutes 18-21% of all Swedish embryo transfers, and frozen-BT has become far more prevalent than frozen-CT [64]. Indeed, a previous study on the CoNARTaS cohort showed a steadily increasing risk of placenta previa after ART compared to NC between 1988 and 2015, which might partly be explained by a shift from cleavage stage to blastocyst embryo transfer [10]. Nevertheless, caution is needed when generalizing our findings to populations outside the Nordic countries, as no clear pattern between placenta previa, embryo culture duration and cryopreservation status was found in a large registry-based study from Japan [27].

## **Conclusions**

Our study indicates that the higher risk of placenta previa after ART is associated with both treatment and maternal factors, where fresh-ET, and especially fresh-BT, was associated with an increased risk of placenta previa. Furthermore, women diagnosed with endometriosis and receiving ART seemed to be at particularly high risk, possibly due to contributions from ART. Further insights into the mechanisms responsible might lead to development of safer ART methods. Although the risk of placenta previa is unlikely to be weighted heavily when choosing the type of ART treatment, it might be considered along with the risk of other and more severe pregnancy complications, especially if the alternative treatment types will not reduce the chances of achieving a pregnancy.

# **Acknowledgements**

Not applicable.

# **References**

1.	Chambers GM, Dyer S, Zegers-Hochschild F, de Mouzon J, Ishihara O, Banker M, et al. International Committee for Monitoring Assisted Reproductive Technologies world report: assisted reproductive technology, 2014†. Hum Reprod. 2021;36: 2921–2934. doi:10.1093/humrep/deab198

2.	Schmidt L, Sobotka T, Bentzen JG, Nyboe Andersen A, ESHRE Reproduction and Society Task Force. Demographic and medical consequences of the postponement of parenthood. Hum Reprod Update. 2012;18: 29–43. doi:10.1093/humupd/dmr040

3.	Smeenk J, Wyns C, The European IVF Monitoring Consortium (EIM) for the European Society of Human Reproduction and Embryology (ESHRE), De Geyter C, Kupka M, Bergh C, et al. ART in Europe, 2019: results generated from European registries by ESHRE†. Hum Reprod. 2023;38: 2321–2338. doi:10.1093/humrep/dead197

4.	Karami M, Jenabi E, Fereidooni B. The association of placenta previa and assisted reproductive techniques: a meta-analysis. J Matern Fetal Neonatal Med. 2018;31: 1940–1947. doi:10.1080/14767058.2017.1332035

5.	Bosdou JK, Anagnostis P, Goulis DG, Lainas GT, Tarlatzis BC, Grimbizis GF, et al. Risk of gestational diabetes mellitus in women achieving singleton pregnancy spontaneously or after ART: a systematic review and meta-analysis. Hum Reprod Update. 2020;26: 514–544. doi:10.1093/humupd/dmaa011

6.	Keukens A, van Wely M, van der Meulen C, Mochtar MH. Pre-eclampsia in pregnancies resulting from oocyte donation, natural conception or IVF: a systematic review and meta-analysis. Hum Reprod. 2022;37: 586–599. doi:10.1093/humrep/deab267

7.	Qin J, Liu X, Sheng X, Wang H, Gao S. Assisted reproductive technology and the risk of pregnancy-related complications and adverse pregnancy outcomes in singleton pregnancies: a meta-analysis of cohort studies. Fertil Steril. 2016;105: 73-85.e1–6. doi:10.1016/j.fertnstert.2015.09.007

8.	Henningsen AA, Gissler M, Skjaerven R, Bergh C, Tiitinen A, Romundstad LB, et al. Trends in perinatal health after assisted reproduction: a Nordic study from the CoNARTaS group. Hum Reprod. 2015;30: 710–716. doi:10.1093/humrep/deu345

9.	Oberg AS, VanderWeele TJ, Almqvist C, Hernandez-Diaz S. Pregnancy complications following fertility treatment-disentangling the role of multiple gestation. Int J Epidemiol. 2018;47: 1333–1342. doi:10.1093/ije/dyy103

10.	Petersen SH, Bergh C, Gissler M, Åsvold BO, Romundstad LB, Tiitinen A, et al. Time trends in placenta-mediated pregnancy complications after assisted reproductive technology in the Nordic countries. Am J Obstet Gynecol. 2020;223: 226.e1-226.e19. doi:10.1016/j.ajog.2020.02.030

11.	Crane JM, Van den Hof MC, Dodds L, Armson BA, Liston R. Maternal complications with placenta previa. Am J Perinatol. 2000;17: 101–105. doi:10.1055/s-2000-9269

12.	Onwere C, Gurol-Urganci I, Cromwell DA, Mahmood TA, Templeton A, van der Meulen JH. Maternal morbidity associated with placenta praevia among women who had elective caesarean section. Eur J Obstet Gynecol Reprod Biol. 2011;159: 62–66. doi:10.1016/j.ejogrb.2011.07.008

13.	Lal AK, Hibbard JU. Placenta previa: an outcome-based cohort study in a contemporary obstetric population. Arch Gynecol Obstet. 2015;292: 299–305. doi:10.1007/s00404-015-3628-y

14.	Fan D, Xia Q, Liu L, Wu S, Tian G, Wang W, et al. The Incidence of Postpartum Hemorrhage in Pregnant Women with Placenta Previa: A Systematic Review and Meta-Analysis. PLoS One. 2017;12: e0170194. doi:10.1371/journal.pone.0170194

15.	Gibbins KJ, Einerson BD, Varner MW, Silver RM. Placenta Previa and Maternal Hemorrhagic Morbidity. J Matern Fetal Neonatal Med. 2018;31: 494–499. doi:10.1080/14767058.2017.1289163

16.	Salihu HM, Li Q, Rouse DJ, Alexander GR. Placenta previa: neonatal death after live births in the United States. Am J Obstet Gynecol. 2003;188: 1305–1309. doi:10.1067/mob.2003.303

17.	Vahanian SA, Lavery JA, Ananth CV, Vintzileos A. Placental implantation abnormalities and risk of preterm delivery: a systematic review and metaanalysis. Am J Obstet Gynecol. 2015;213: S78-90. doi:10.1016/j.ajog.2015.05.058

18.	Healy DL, Breheny S, Halliday J, Jaques A, Rushford D, Garrett C, et al. Prevalence and risk factors for obstetric haemorrhage in 6730 singleton births after assisted reproductive technology in Victoria Australia. Hum Reprod. 2010;25: 265–274. doi:10.1093/humrep/dep376

19.	Sazonova A, Källen K, Thurin-Kjellberg A, Wennerholm U-B, Bergh C. Factors affecting obstetric outcome of singletons born after IVF. Hum Reprod. 2011;26: 2878–2886. doi:10.1093/humrep/der241

20.	Fernando D, Halliday JL, Breheny S, Healy DL. Outcomes of singleton births after blastocyst versus nonblastocyst transfer in assisted reproductive technology. Fertil Steril. 2012;97: 579–584. doi:10.1016/j.fertnstert.2011.12.032

21.	Ginström Ernstad E, Bergh C, Khatibi A, Källén KBM, Westlander G, Nilsson S, et al. Neonatal and maternal outcome after blastocyst transfer: a population-based registry study. Am J Obstet Gynecol. 2016;214: 378.e1-378.e10. doi:10.1016/j.ajog.2015.12.040

22.	Ginström Ernstad E, Spangmose AL, Opdahl S, Henningsen A-KA, Romundstad LB, Tiitinen A, et al. Perinatal and maternal outcome after vitrification of blastocysts: a Nordic study in singletons from the CoNARTaS group. Hum Reprod. 2019;34: 2282–2289. doi:10.1093/humrep/dez212

23.	Spangmose AL, Ginström Ernstad E, Malchau S, Forman J, Tiitinen A, Gissler M, et al. Obstetric and perinatal risks in 4601 singletons and 884 twins conceived after fresh blastocyst transfers: a Nordic study from the CoNARTaS group. Hum Reprod. 2020;35: 805–815. doi:10.1093/humrep/deaa032

24.	Korosec S, Ban Frangez H, Verdenik I, Kladnik U, Kotar V, Virant-Klun I, et al. Singleton Pregnancy Outcomes after In Vitro Fertilization with Fresh or Frozen-Thawed Embryo Transfer and Incidence of Placenta Praevia. Biomed Res Int. 2014;2014: 431797. doi:10.1155/2014/431797

25.	Volodarsky-Perel A, Ton Nu TN, Buckett W, Machado-Gedeon A, Cui Y, Shaul J, et al. Effect of embryo stage at transfer on placental histopathology features in singleton live births resulting from fresh embryo transfers. Fertil Steril. 2021;115: 673–682. doi:10.1016/j.fertnstert.2020.06.017

26.	Cirillo F, Grilli L, Ronchetti C, Paladino I, Morenghi E, Busnelli A, et al. Retrospective comparison of pregnancy outcomes of fresh and frozen-warmed single blastocyst transfer: a 5-year single-center experience. J Assisted Reprod Genet. 2022;39: 201–209. doi:10.1007/s10815-021-02362-3

27.	Ishihara O, Araki R, Kuwahara A, Itakura A, Saito H, Adamson GD. Impact of frozen-thawed single-blastocyst transfer on maternal and neonatal outcome: an analysis of 277,042 single-embryo transfer cycles from 2008 to 2010 in Japan. Fertil Steril. 2014;101: 128–133. doi:10.1016/j.fertnstert.2013.09.025

28.	Wei D, Liu J-Y, Sun Y, Shi Y, Zhang B, Liu J-Q, et al. Frozen versus fresh single blastocyst transfer in ovulatory women: a multicentre, randomised controlled trial. Lancet. 2019;393: 1310–1318. doi:10.1016/S0140-6736(18)32843-5

29.	Wang J, Liu Q, Deng B, Chen F, Liu X, Cheng J. Pregnancy outcomes of Chinese women undergoing IVF with embryonic cryopreservation as compared to natural conception. BMC Pregnancy Childbirth. 2021;21: 39. doi:10.1186/s12884-020-03486-7

30.	Carusi DA, Gopal D, Cabral HJ, Bormann CL, Racowsky C, Stern JE. A unique placenta previa risk factor profile for pregnancies conceived with assisted reproductive technology. Fertil Steril. 2022;118: 894–903. doi:10.1016/j.fertnstert.2022.08.013

31.	Frisell T. Invited Commentary: Sibling-Comparison Designs, Are They Worth the Effort? Am J Epidemiol. 2021;190: 738–741. doi:10.1093/aje/kwaa183

32.	Lawlor DA, Tilling K, Davey Smith G. Triangulation in aetiological epidemiology. Int J Epidemiol. 2016;45: 1866–1886. doi:10.1093/ije/dyw314

33.	Opdahl S, Henningsen A-KA, Bergh C, Gissler M, Romundstad LB, Petzold M, et al. Data Resource Profile: Committee of Nordic Assisted Reproductive Technology and Safety (CoNARTaS) cohort. Int J Epidemiol. 2020;49: 365–366f. doi:10.1093/ije/dyz228

34.	Zegers-Hochschild F, Adamson GD, Dyer S, Racowsky C, de Mouzon J, Sokol R, et al. The International Glossary on Infertility and Fertility Care, 2017. Fertil Steril. 2017;108: 393–406. doi:10.1016/j.fertnstert.2017.06.005

35.	Jansen CHJR, Kleinrouweler CE, van Leeuwen L, Ruiter L, Limpens J, van Wely M, et al. Final outcome of a second trimester low-positioned placenta: A systematic review and meta-analysis. Eur J Obstet Gynecol Reprod Biol. 2019;240: 197–204. doi:10.1016/j.ejogrb.2019.06.020

36.	Grønbeck L, Andersen LLT, Nørgaard LN. Placenta prævia og abnorm invasiv placenta. Dansk Selskab for Obstetrik og Gynækologi; 2017. Available: https://www.dsog.dk/s/16112019_Placenta-prvia.pdf

37.	Bolin K, Varli IH, Wollmann CL, Svanvik T, Skånes LT. SFOG-råd Handläggning av graviditet och förlossning vid placenta previa, vasa previa och invasiv placenta (accreta). Swedish Society of Obstetrics and Gynecology; 2020. Available: https://www.sfog.se/media/337261/sfog-ra-d-placenta-previa-vasa-previa-och-invasiv-placenta-accreta-_slutgiltig_201215.pdf

38.	Nyfløt LT, Pettersen S, Ebbing C, Almås I, Husby H, Aase TA, et al. Invasiv placenta, placenta previa og vasa previa. Norsk gynekologisk forening; 2020. Available: https://www.legeforeningen.no/foreningsledd/fagmed/norsk-gynekologisk-forening/veiledere/veileder-i-fodselshjelp/invasiv-placenta-placenta-previa-og-vasa-previa/

39.	Hernán MA, Robins JM. 3.3- Positivity. Causal inference: What If. Boca Raton: Chapman & Hall/CRC; 2020. pp. 32–33. Available: https://www.hsph.harvard.edu/miguel-hernan/wp-content/uploads/sites/1268/2024/01/hernanrobins_WhatIf_2jan24.pdf

40.	Jenabi E, Salimi Z, Bashirian S, Khazaei S, Ayubi E. The risk factors associated with placenta previa: An umbrella review. Placenta. 2022;117: 21–27. doi:10.1016/j.placenta.2021.10.009

41.	Benaglia L, Candotti G, Papaleo E, Pagliardini L, Leonardi M, Reschini M, et al. Pregnancy outcome in women with endometriosis achieving pregnancy with IVF. Hum Reprod. 2016;31: 2730–2736. doi:10.1093/humrep/dew210

42.	Ibiebele I, Nippita T, Baber R, Torvaldsen S. Pregnancy outcomes in women with endometriosis and/or ART use: a population-based cohort study. Hum Reprod. 2022;37: 2350–2358. doi:10.1093/humrep/deac186

43.	Gómez-Pereira E, Burgos J, Mendoza R, Pérez-Ruiz I, Olaso F, García D, et al. Endometriosis Increases the Risk of Placenta Previa in Both IVF Pregnancies and the General Obstetric Population. Reprod Sci. 2023;30: 854–864. doi:10.1007/s43032-022-01054-2

44.	Romundstad LB, Romundstad PR, Sunde A, von Düring V, Skjaerven R, Vatten LJ. Increased risk of placenta previa in pregnancies following IVF/ICSI; a comparison of ART and non-ART pregnancies in the same mother. Hum Reprod. 2006;21: 2353–2358. doi:10.1093/humrep/del153

45.	Baba K, Ishihara O, Hayashi N, Saitoh M, Taya J, Kinoshita K. Where does the embryo implant after embryo transfer in humans? Fertil Steril. 2000;73: 123–125. doi:10.1016/s0015-0282(99)00454-9

46.	Knutzen V, Stratton CJ, Sher G, McNamee PI, Huang TT, Soto-Albors C. Mock embryo transfer in early luteal phase, the cycle before in vitro fertilization and embryo transfer: a descriptive study. Fertil Steril. 1992;57: 156–162. doi:10.1016/S0015-0282(16)54793-1

47.	Rombauts L, Motteram C, Berkowitz E, Fernando S. Risk of placenta praevia is linked to endometrial thickness in a retrospective cohort study of 4537 singleton assisted reproduction technology births. Hum Reprod. 2014;29: 2787–2793. doi:10.1093/humrep/deu240

48.	Kalakota NR, George LC, Morelli SS, Douglas NC, Babwah AV. Towards an Improved Understanding of the Effects of Elevated Progesterone Levels on Human Endometrial Receptivity and Oocyte/Embryo Quality during Assisted Reproductive Technologies. Cells. 2022;11: 1405. doi:10.3390/cells11091405

49.	Bourgain C, Ubaldi F, Tavaniotou A, Smitz J, Van Steirteghem AC, Devroey P. Endometrial hormone receptors and proliferation index in the periovulatory phase of stimulated embryo transfer cycles in comparison with natural cycles and relation to clinical pregnancy outcome. Fertil Steril. 2002;78: 237–244. doi:10.1016/S0015-0282(02)03228-4

50.	Oron G, Hiersch L, Rona S, Prag-Rosenberg R, Sapir O, Tuttnauer-Hamburger M, et al. Endometrial thickness of less than 7.5 mm is associated with obstetric complications in fresh IVF cycles: a retrospective cohort study. Reprod Biomed Online. 2018;37: 341–348. doi:10.1016/j.rbmo.2018.05.013

51.	Guo Z, Xu X, Zhang L, Zhang L, Yan L, Ma J. Endometrial thickness is associated with incidence of small-for-gestational-age infants in fresh in vitro fertilization–intracytoplasmic sperm injection and embryo transfer cycles. Fertil Steril. 2020;113: 745–752. doi:10.1016/j.fertnstert.2019.12.014

52.	Jing S, Li X, Zhang S, Gong F, Lu G, Lin G. The risk of placenta previa and cesarean section associated with a thin endometrial thickness: a retrospective study of 5251 singleton births during frozen embryo transfer in China. Arch Gynecol Obstet. 2019;300: 1227–1237. doi:10.1007/s00404-019-05295-6

53.	Zhang X, Liang L, Hu Y, Zhang Z, Wu Y, Zhang X, et al. A thinner endometrium is associated with lower newborn birth weight during *in vitro* fertilization–frozen-embryo transfer: a cohort study. Gynecol Endocrinol. 2023;39: 2217269. doi:10.1080/09513590.2023.2217269

54.	Zhu H, Liu L, Yang L, Xue Y, Tong X, Jiang L, et al. The effect of progesterone level prior to oocyte retrieval on the numbers of oocytes retrieved and embryo quality in IVF treatment cycles: an analysis of 2,978 cycles. J Assist Reprod Genet. 2014;31: 1183–1187. doi:10.1007/s10815-014-0291-3

55.	Magnusson Å, Wennerholm U-B, Källén K, Petzold M, Thurin- Kjellberg A, Bergh C. The association between the number of oocytes retrieved for IVF, perinatal outcome and obstetric complications. Hum Reprod. 2018;33: 1939–1947. doi:10.1093/humrep/dey266

56.	Rabe-Hesketh S, Skrondal A. 10.14.1- Conditional logistic regression. Multilevel and longitudinal modeling using Stata- Volume II: Categorical Responses, Counts, and Survival- Third Edition. STATA press; 2012. pp. 557–559.

57.	Bi S, Zhang L, Wang Z, Chen J, Tang J, Gong J, et al. Effect of types of placenta previa on maternal and neonatal outcomes: a 10-year retrospective cohort study. Arch Gynecol Obstet. 2021;304: 65–72. doi:10.1007/s00404-020-05912-9

58.	Jansen CHJR, van Dijk CE, Kleinrouweler CE, Holzscherer JJ, Smits AC, Limpens JCEJM, et al. Risk of preterm birth for placenta previa or low-lying placenta and possible preventive interventions: A systematic review and meta-analysis. Front Endocrinol (Lausanne). 2022;13: 921220. doi:10.3389/fendo.2022.921220

59.	Jauniaux E, Alfirevic Z, Bhide A, Belfort M, Burton G, Collins S, et al. Placenta Praevia and Placenta Accreta: Diagnosis and Management. BJOG. 2019;126: e1–e48. doi:10.1111/1471-0528.15306

60.	Ludvigsson JF, Andersson E, Ekbom A, Feychting M, Kim J-L, Reuterwall C, et al. External review and validation of the Swedish national inpatient register. BMC Public Health. 2011;11: 1–16. doi:10.1186/1471-2458-11-450

61.	Penzias A, Bendikson K, Falcone T, Hansen K, Hill M, Jindal S, et al. Evidence-based treatments for couples with unexplained infertility: a guideline. Fertil Steril. 2020;113: 305–322. doi:10.1016/j.fertnstert.2019.10.014

62.	Matsuzaki S, Nagase Y, Ueda Y, Kakuda M, Maeda M, Matsuzaki S, et al. Placenta Previa Complicated with Endometriosis: Contemporary Clinical Management, Molecular Mechanisms, and Future Research Opportunities. Biomedicines. 2021;9: 1536. doi:10.3390/biomedicines9111536

63.	Velez MP, Bougie O, Bahta L, Pudwell J, Griffiths R, Li W, et al. Mode of conception in patients with endometriosis and adverse pregnancy outcomes: a population-based cohort study. Fertil Steril. 2022;118: 1090–1099. doi:10.1016/j.fertnstert.2022.09.015

64.	Q-IVF. National reports from Q-IVF (Nationellt kvalitetsregister för assisterad befruktning). 2023 [cited 12 Dec 2024]. Available: https://www.medscinet.com/qivf/arsrapporter.aspx

# **Supporting information captions**

**S1 Table. Data sources and registration practice for placenta previa in the Nordic countries during the study period.** ^a^ Open text field where the reporter was free to put ICD-codes or plain text. In the case of plain text, data were recoded as ICD-codes by the registry holder.

**S2 Table. Data sources and registration practice for infertility factors in the Nordic countries during the study period.** ^a^ In Norway, the National Patient Registry only provided diagnoses of polycystic ovary syndrome.

**S3 Table.** ICD codes used for identification of infertility factors in the Nordic countries during the study period.

**S1 Text. More detailed description of sensitivity analyses.**

**S1 Checklist.**

**S1 Study Protocol.**

**S4 Table.** **Observed risk and adjusted odds ratio of placenta previa in second pregnancy according to conception method and caesarean section in first pregnancy in sample 1.** Abbreviations: ART, assisted reproductive technology; NC, natural conception; CI, confidence interval. ^a^ Results are presented for the full study period. ^b^ Observed proportions are presented for mothers with at least five years of follow-up after first delivery. ^c^ Adjusted for year of delivery (categorical: 1988–1996, 1997–2001, 2002–2006, 2007–2011, 2012–2015), maternal age (categorical: 20–24, 25–29, 30–34, 35–39, 40–45), parity (categorical: 0, 1, 2, 3) and country.

**S5 Table. Observed risk and adjusted odds ratio of placenta previa in second pregnancy according to conception method and placenta previa in first pregnancy in sample 1.** Abbreviations: ART, assisted reproductive technology; NC, natural conception; CI, confidence interval. ^a^ Results are presented for the full study period. ^b^ Observed proportions are presented for mothers with at least five years of follow-up after first delivery. ^c^ Adjusted for year of delivery (categorical: 1988–1996, 1997–2001, 2002–2006, 2007–2011, 2012–2015), maternal age (categorical: 20–24, 25–29, 30–34, 35–39, 40–45), parity (categorical: 0, 1, 2, 3) and country.

**S6 Table. Description of pregnancies with and without placenta previa according to conception method and plurality in sample 1.** Abbreviations: ART, assisted reproductive technology; NC, natural conception; Previa, Placenta previa; SD, standard deviation; SGA, small for gestational age; LGA, large for gestational age. ^a^ Gestational age <37 weeks. ^b^ Gestational age <32 weeks. ^c^ Data on birthweight were available for >99.2% in each category. ^d^ Birthweight z-score is defined as number of standard deviations from expected birthweight according to gestational age in days and sex. It was calculated using Marsal’s formulas, with one standard deviation equal to 11% [1]. ^e^ Birthweight z-score <-2. ^f^ Birthweight z-score >2.
